# Structure-based engineering of substrate specificity for pinoresinol-lariciresinol reductases

**DOI:** 10.1038/s41467-021-23095-y

**Published:** 2021-05-14

**Authors:** Ying Xiao, Kai Shao, Jingwen Zhou, Lian Wang, Xueqi Ma, Di Wu, Yingbo Yang, Junfeng Chen, Jingxian Feng, Shi Qiu, Zongyou Lv, Lei Zhang, Peng Zhang, Wansheng Chen

**Affiliations:** 1 Research and Development Center of Chinese Medicine Resources and Biotechnology, Institute of Chinese Materia Medica, Shanghai University of Traditional Chinese Medicine, Shanghai, China; 2 National Key Laboratory of Plant Molecular Genetics, CAS Center for Excellence in Molecular Plant Sciences, Institute of Plant Physiology and Ecology, Chinese Academy of Sciences, Shanghai, China; 3 University of Chinese Academy of Sciences, Beijing, China; 4 National Engineering Laboratory for Cereal Fermentation Technology and School of Biotechnology, Jiangnan University, Wuxi, Jiangsu China; 5 Science Center for Future Foods, Jiangnan University, Wuxi, Jiangsu China; 6 Department of Pharmaceutical Botany, School of Pharmacy, Naval Medical University (Second Military Medical University), Shanghai, China; 7 Department of Pharmacy, Changzheng Hospital, Naval Medical University (Second Military Medical University), Shanghai, China

**Keywords:** Enzyme mechanisms, Secondary metabolism, X-ray crystallography

## Abstract

Pinoresinol–lariciresinol reductases (PLRs) are enzymes involved in the lignan biosynthesis after the initial dimerization of two monolignols, and this represents the entry point for the synthesis of 8-8′ lignans and contributes greatly to their structural diversity. Of particular interest has been the determination of how differing substrate specificities are achieved with these enzymes. Here, we present crystal structures of *Ii*PLR1 from *Isatis indigotica* and pinoresinol reductases (PrRs) *At*PrR1 and *At*PrR2 from *Arabidopsis thaliana*, in the apo, substrate-bound and product-bound states. Each structure contains a head-to-tail homodimer, and the catalytic pocket comprises structural elements from both monomers. β4 loop covers the top of the pocket, and residue 98 from the loop governs catalytic specificity. The substrate specificities of *Ii*PLR1 and *At*PrR2 can be switched via structure-guided mutagenesis. Our study provides insight into the molecular mechanism underlying the substrate specificity of PLRs/PrRs and suggests an efficient strategy for the large-scale commercial production of the pharmaceutically valuable compound lariciresinol.

## Abstract (web summary)

Pinoresinol–lariciresinol reductases (PLRs) are enzymes involved in the lignan biosynthesis. Here, crystal structures of three PLRs in the apo, substrate-bound and product-bound states, and accompanying mutagenesis provide insight into PLRs catalytic mechanism and suggest a strategy for PLR engineering.

## Introduction

Lignans are a major group of secondary metabolites in plants^1^. This family has numerous biological effects in humans (e.g., anticancer^2^, antiviral^3^, antioxidant^4^, and immunosuppression^5^) owing to their structural diversity—nearly 2000 distinct lignans have been reported. For example, the furofuran lignans such as kandelisesquilignan A/B and terminaloside K have antioxidant effects^6,7^. The dibenzylbutyrolactone lignans including arctigenin, traxillagenin, arctiin, traxillaside, and their glycosides have neuroprotective activities^8^. Finally, the aryltetralin lignan podophyllotoxin^9^ is the precursor for the semi-synthesis of anticancer drugs such as etoposide^10^.

Lignans biosynthesis starts with the coupling of two coniferyl alcohols by an oxidase (laccase or peroxidase) with the aid of a dirigent protein to form pinoresinol^11^. Pinoresinol/lariciresinol reductase (PLR), an NADPH-dependent reductase, converts pinoresinol to lariciresinol and subsequently to secoisolariciresinol^12^. Because the reductive steps that give rise to lariciresinol and secoisolariciresinol represent entry points for the biosynthesis of the lignan subclasses furofurans, dibenzylbutane, dibenzylbutyrolactone, and aryltetrahydronaphthalene^13^, PLR is regarded as a pivotal enzyme that contributes to lignan structural diversity. Moreover, variation in both the composition and accumulation of lignans among different plant species, organs, and developmental stages can be ascribed, at least in part, to the characteristics of reactions catalyzed by PLRs as well as their expression patterns^14^. Therefore, characterization of the catalytic mechanisms of PLRs—especially their substrate selectivity—is particularly crucial for understanding the molecular basis of the remarkable diversity of both chemical structures and biological activities of lignans.

The substrate selectivity of PLRs has attracted considerable attention^12^. Most PLRs that have been characterized reduce both pinoresinol and lariciresinol efficiently to produce lariciresinol and secoisolariciresinol, respectively^12^. Known exceptions are *Arabidopsis thaliana* reductases that have substrate preference for pinoresinol, but only weak (*At*PrR1) or no activity (*At*PrR2) toward lariciresinol and are thus named pinoresinol reductases (PrRs)^15^. A recent study indicated that the L174I mutant of *Camellia sinensis* PLR1 (*Cs*PLR1) loses the capacity to reduce pinoresinol and specifically catalyzes the conversion of lariciresinol to secoisolariciresinol, but the underlying mechanism is unclear^16^. The three-dimensional structure of *Thuja plicata* PLR1 (*Tp*PLR1) has been elucidated and indicates that K138 is responsible for the basic catalysis, because the mutant K138A lacks the ability to convert pinoresinol^17^. However, the apo structure does not provide sufficient information to interpret the substrate-selective mechanism of PLRs/PrRs.

**Fig. 1:**
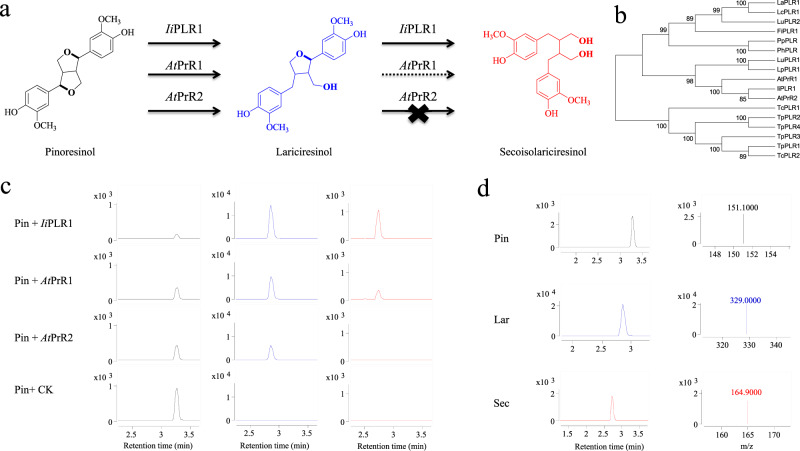
Biochemical assays for *Ii*PLR1 and *At*PrRs function. **a** *Ii*PLR1 efficiently catalyzes the conversion of pinoresinol into lariciresinol and also catalyzes the conversion of lariciresinol into secoisolariciresinol. In contrast, *At*PrR1/2 exhibit a substrate preference for pinoresinol, yet exhibit only weak activity (*At*PrR1) or no activity (*At*PrR2) for lariciresinol. **b** Phylogenetic tree of PLRs/PrRs from different species. **c** Conversion of pinoresinol into lariciresinol and then into secoisolariciresinol by recombinant *Ii*PLR1, *At*PrR1, and *At*PrR2. The reaction products were analyzed by LC-MS. **d** Chromatograms for pinoresinol, lariciresinol, and secoisolariciresinol are denoted in black, blue, and red, respectively.

Phylogenetic analysis of PLRs/PrRs from different species has revealed that *Isatis indigotica* PLR1 (*Ii*PLR1), a key enzyme involved in lariciresinol biosynthesis, has the closest relationship to *At*PrRs with a high level of amino-acid sequence identity (>80%)^18^ and is grouped with *At*PrR2, which cannot utilize lariciresinol as substrate (Fig. 1). Interestingly, in contrast to *At*PrRs that has substrate preference for pinoresinol, *Ii*PLR1 from *I. indigotica* (family Cruciferae, same as *A. thaliana*) can reduce both pinoresinol and lariciresinol efficiently with comparable *k*_cat_*/K*_m_ values^18^. The finding that *Ii*PLR1/*At*PrRs, which differ in substrate selectivity, are clustered together suggests that substrate specificity is independent of sequence conservation among PLRs/PrRs. Therefore, the amino-acid residues responsible for PLR/PrR substrate selectivity are difficult to determine merely through sequence analysis, and thus structural information on PLR/PrR enzymes is vital—as are data concerning how these two enzyme types can utilize two different substrates.

In the present work, we compare crystal structures of *Ii*PLR1, *At*PrR1, and *At*PrR2 and identify residues that may be responsible for the observed substrate selectivity of PLRs and PrRs. Mutagenesis of these residues alters the substrate specificities for pinoresinol and lariciresinol. For example, mutagenesis of *Ii*PLR1 successfully eliminates the second reaction that converts lariciresinol to secoisolariciresinol, leading to a high accumulation of the pharmaceutically valuable compound lariciresinol. Our study will enable the synthesis of lignans with diverse chemical structures and bioactivities by biotechnological means or by enzyme-assisted chemistry.

## Results

### Characterization of *Ii*PLR1, *At*PrR1, and *At*PrR2 crystal structures

**Fig. 2:**
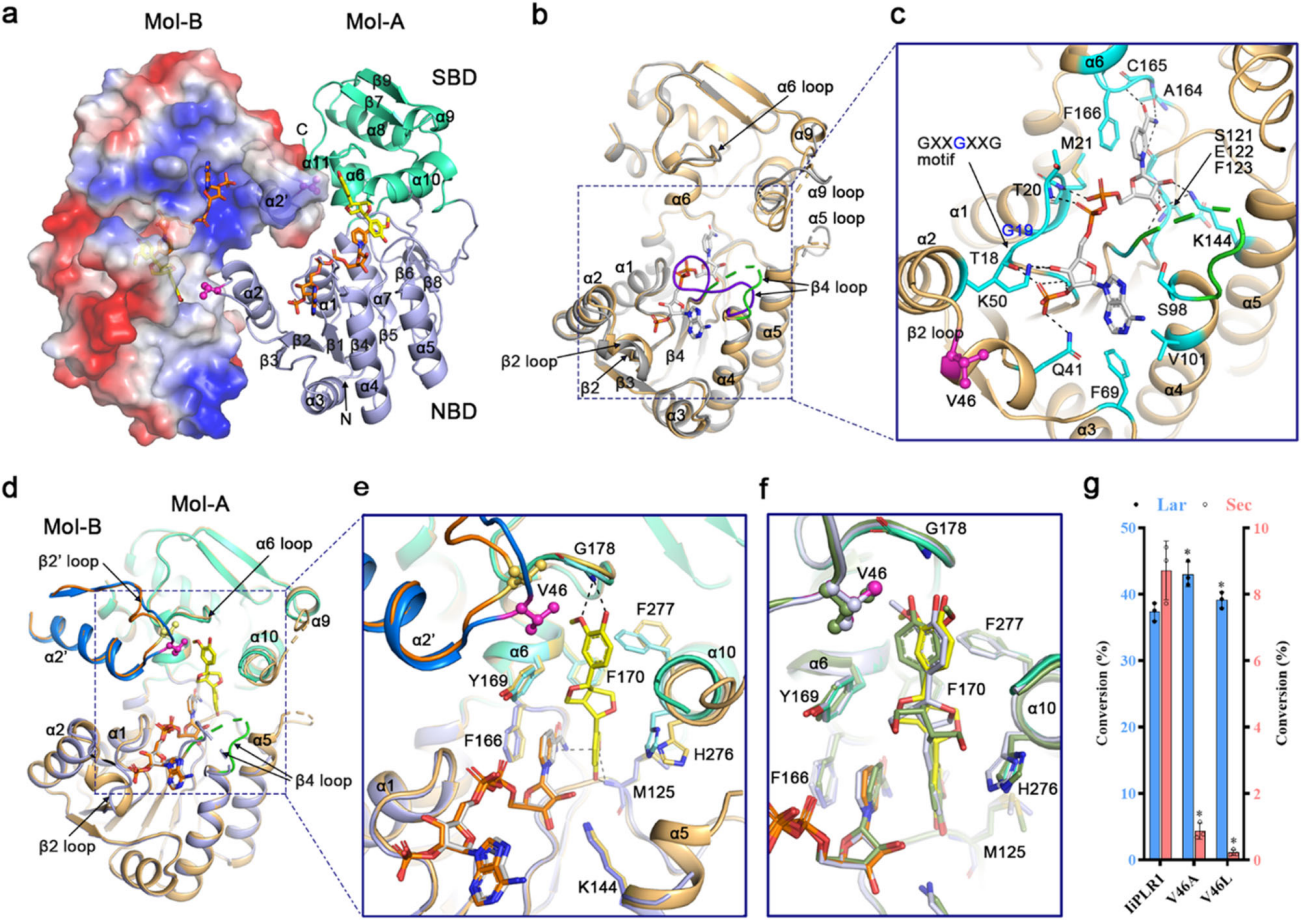
Structural mechanism of continuous catalytic reactions by *Ii*PLR1 based on homodimerization. **a** Dimer formation of *Ii*PLR1_NAP_ + PIN. Mol-A is shown as cartoon model, with its NBD and SBD colored in light blue and green cyan, respectively. Mol-B is represented as an electrostatic-surface model, on which blue and red colors represent positive and negative charges, respectively. NADP^+^ and (+)-pinoresinol are shown as sticks and colored in orange and yellow, respectively. **b** Conformational changes were assessed by comparing monomer structures of *Ii*PLR1_apo (gray) and *Ii*PLR1_NAP (light orange). The NADP^+^ bound to *Ii*PLR1_NAP is colored gray. The β4 loops of *Ii*PLR1_apo and *Ii*PLR1_NAP are highlighted as purple and green, respectively. **c** Zoom-in view of the NADPH-binding groove of *Ii*PLR1_NAP. Residues interacting with NADP^+^ are colored cyan. The conserved GXXGXXG motif is indicated. Residue Val46 involved in dimer formation and substrate binding is shown as a ball-and-stick model and colored magenta. Dotted lines denote possible hydrogen bonds. **d** Structure comparison of *Ii*PLR1_NAP_ + PIN and *Ii*PLR1_NAP. Mol-Bs of *Ii*PLR1_NAP_ + PIN and *Ii*PLR1_NAP, Val46s in *Ii*PLR1_NAP_ + PIN, and *Ii*PLR1_NAP are colored in marine, orange, magenta, and light yellow, respectively. **e** Zoom-in view of the substrate-binding groove. Residues of Mol-As in *Ii*PLR1_NAP_ + PIN and *Ii*PLR1_NAP are colored green cyan (or light blue) and light yellow, respectively. **f** Structural comparation of the substrate/product-binding grooves in *Ii*PLR1_NAP_ + PIN, *Ii*PLR1_NAP_-LAR (blue white), and *Ii*PLR1_NAP_-SEC (smudge). The cartoons are generated by PyMOL. **g** Enzyme assays for wild-type *Ii*PLR1 and its mutants V46A and V46L. Data are mean±s.d. (*n* = 3 independent experiments). Asterisk * indicates significant difference from the wild-type enzyme (*P* < 0.05) analyzed by one-way ANOVA with Tukey’s multiple comparisons test. Source data underlying Fig. 2g are provided as a Source Data file.

To understand both the catalytic mechanism of PLR and the mechanism underlying the substrate specificity of PLRs/PrRs, we chose *Ii*PLR1, *At*PrR1, and *At*PrR2 for a structure study. The crystal structures were captured in the apo, substrate-bound and/or product-bound forms (Table 1). We found that, for all 16 structures we solved, each enzyme adopts a similar head-to-tail dimer conformation (Fig. 2a and Supplementary Fig. 1), strongly suggesting that each PLR/PrR functions as a homodimer, consistent with the literature that *Tp*PLR1 exists as a dimeric entity in solution^17^.

**Table 1:** The crystal structure information of *Ii*PLR1, *At*PrR1, and *At*PrR2 proteins in their apo, NADP^+^ and substrate/product binding forms.

	apo	NADP^+^	NADP^+^		NADP^+^		NADP^+^	
			(+)-Pinoresinol	(−)-Pinoresinol	(+)-Lariciresinol	(−)-Lariciresinol	(+)-Secoisolariciresinol	(−)-Secoisolariciresinol
*Ii*PLR1	*Ii*PLR_apo 2.7 Å	*Ii*PLR1_NAP 2.4 Å	*Ii*PLR1_NAP_ + PIN 2.3 Å	*Ii*PLR1_NAP_-PIN 2.1 Å	–	*Ii*PLR1_NAP_-LAR 2.2 Å	*Ii*PLR1_NAP_ + SEC 2.3 Å	*Ii*PLR1_NAP_-SEC 2.6 Å
*At*PrR1	*At*PrR1_apo 2.8 Å	*At*PrR1_NAP 2.0 Å	*At*PrR1_NAP_ + PIN 2.0 Å	*At*PrR1_NAP_-PIN 2.5 Å	*At*PrR1_NAP_ + LAR 1.8 Å	*At*PrR1_NAP_-LAR 2.5 Å	–	*At*PrR1_NAP_-SEC 2.0 Å
*At*PrR2	*At*PrR2_apo 2.0 Å	–	*At*PrR2_NAP_ + PIN 1.6 Å	–	–	–	–	–

Taking the structure of *Ii*PLR1_NAP_+PIN for the purpose of a detailed description, each protomer contains two domains, namely the N-terminal NADPH binding domain (NBD) and the C-terminal substrate binding domain (SBD). The NBD comprises seven β-strands (β1−6, β8) surrounded by six α-helices (α1−5, α7), whereas the SBD comprises two β-strands (β7, β9) with five small α-helices (α6, α8−11). A large groove is formed between NBD and SBD (Fig. 2a). This groove can be roughly divided into two parts—the positively charged part that associates with the NBD and the hydrophobic part that associates with the SBD. The substrates or products can be clearly defined within the groove (Fig. 2a and Supplementary Fig. 2). Several regions (α5 loop, α9-helix, and α9 loop) are partially disordered both in the *Ii*PLR1_apo and *Ii*PLR1_NAP structures, for which the differences can be characterized by a RMSD of 0.287 Å, whereas the β4 loop is well defined in the apo structure (Fig. 2b). Intriguingly, the β4 loop is well defined in the *At*PrR1_NAP structure but disordered in the *At*PrR1/2_apo structures (Supplementary Fig. 3). These structural differences suggest that the loops are somewhat flexible, and act as a switch to control the binding of NADPH and release of NADP^+^. Moreover, the β2 loop moves slightly towards NADP^+^ in the *Ii*PLR1_NAP structure, catering to the entry of the coenzyme (Fig. 2b). NADP^+^ forms strong hydrogen bonds and hydrophobic interactions with residues within the groove (Fig. 2c), among which the GXXGXXG motif (considered as the conserved NADPH-binding motif) binds the phosphate and deoxyribose groups, and residues Ala164, Cys165 together with Phe166 fix the position of the catalytically active nicotinamide group. Residue Lys144, which corresponds to the previously reported Lys138 in *Tp*PLR1 that serves as the general base for catalysis, forms direct hydrogen bonds with NADP^+^ in *Ii*PLR1_apo and *Ii*PLR1_NAP.

### Catalytic mechanism of PLR based on its homodimeration

The dimers of *Ii*PLR1_NAP and *Ii*PLR1_NAP_ + PIN have similar structures, as suggested by a RMSD of 0.362 Å, indeed, even the NADP^+^ moieties could be aligned almost in the same position (Fig. 2d). Both β4 loops are disordered, further implying their flexibility, but β2 loop and α10-helix from neighboring molecules of *Ii*PLR1_NAP_ + PIN dimer make contacts with and stabilize the substrate (Fig. 2d, e). Similar conformational changes of β2 loop and α10-helix can be seen by comparing the structure of *At*PrR1_NAP_ + PIN with that of *At*PrR1_NAP and the structure of *At*PrR2_NAP_ + PIN with that of *At*PrR2_apo (Supplementary Fig. 4a, b). (+)-Pinoresinol is inserted as a straight chain deep into the hydrophobic groove, for which the hydrophilic ends are stabilized through the formation of hydrogen bonds with main-chain atoms of Met125 and Gly178, and the hydrophobic region is surrounded by a series of hydrophobic groups (Fig. 2e). The inner 2-methoxy-phenol group of (+)-pinoresinol forms a sandwich-like π–π stack comprising the nicotinamide head of NADP^+^ and Phe166. Two furan rings in the middle are surrounded by Tyr169 and Phe170 from α6-helix and by His276 and Phe277 from α10-helix. The outer 2-methoxy-phenol group is coordinated by Phe277 and Val46 of β2 loop from a neighboring protomer, which is distant from the NADP^+^ and (+)-pinoresinol of the protomer (Fig. 2a, c, e). Further, Lys144 is far away from the furan rings, indicating that it may not participate in catalysis directly. Based on this structure analysis, we propose that both the entry and exit of NADPH are controlled by the β4 loop of *Ii*PLR1. Once one molecule of (+)-pinoresinol is captured by the narrow hydrophobic groove, each protomer forces the prepositioning of the α10-helix and β2 loop in the other protomer, resulting in tight binding of the substrate. This allows H: transfer from the NADPH to the proximal furan ring of the substrate to produce one molecule of (+)-lariciresinol.

Regarding the mechanism of the second catalytic step, we further compared the structures of *Ii*PLR1_NAP_ + PIN, *Ii*PLR1_NAP_-LAR and *Ii*PLR1_NAP_-SEC, which revealed a similar mode for substrate/product binding (Fig. 2f). Furthermore, Leu46 (corresponding to Val46 in *Ii*PLR1), His276 and Phe277 of *At*PrR1 are positioned similar to the corresponding residues of *Ii*PLR1 to effect substrate binding or product release, except that the β4 loops cover the substrate/product, which are disordered in the *Ii*PLR1 structures (Fig. 2d and Supplementary Fig. 4a, c, d). The importance of Val46 for catalysis in *Ii*PLR1 is underscored by data from a mutational analysis (Fig. 2g). Mutation of Val46 to Ala improved the conversion of pinoresinol to lariciresinol by ~16%, and the subsequent conversion to secoisolariciresinol was greatly reduced. The *Ii*PLR1 mutant V46L had little ability to catalyze the conversion of lariciresinol to secoisolariciresinol. These data suggest that *Ii*PLR1 undergoes substrate-induced conformational changes upon homodimerization to achieve catalysis, and the principle of catalytic reactions using lariciresinol as substrate (second step) appears to be like that using pinoresinol as substrate (first step).

### Mechanism underlying the substrate selectivity of PLR/PrR

**Fig. 3:**
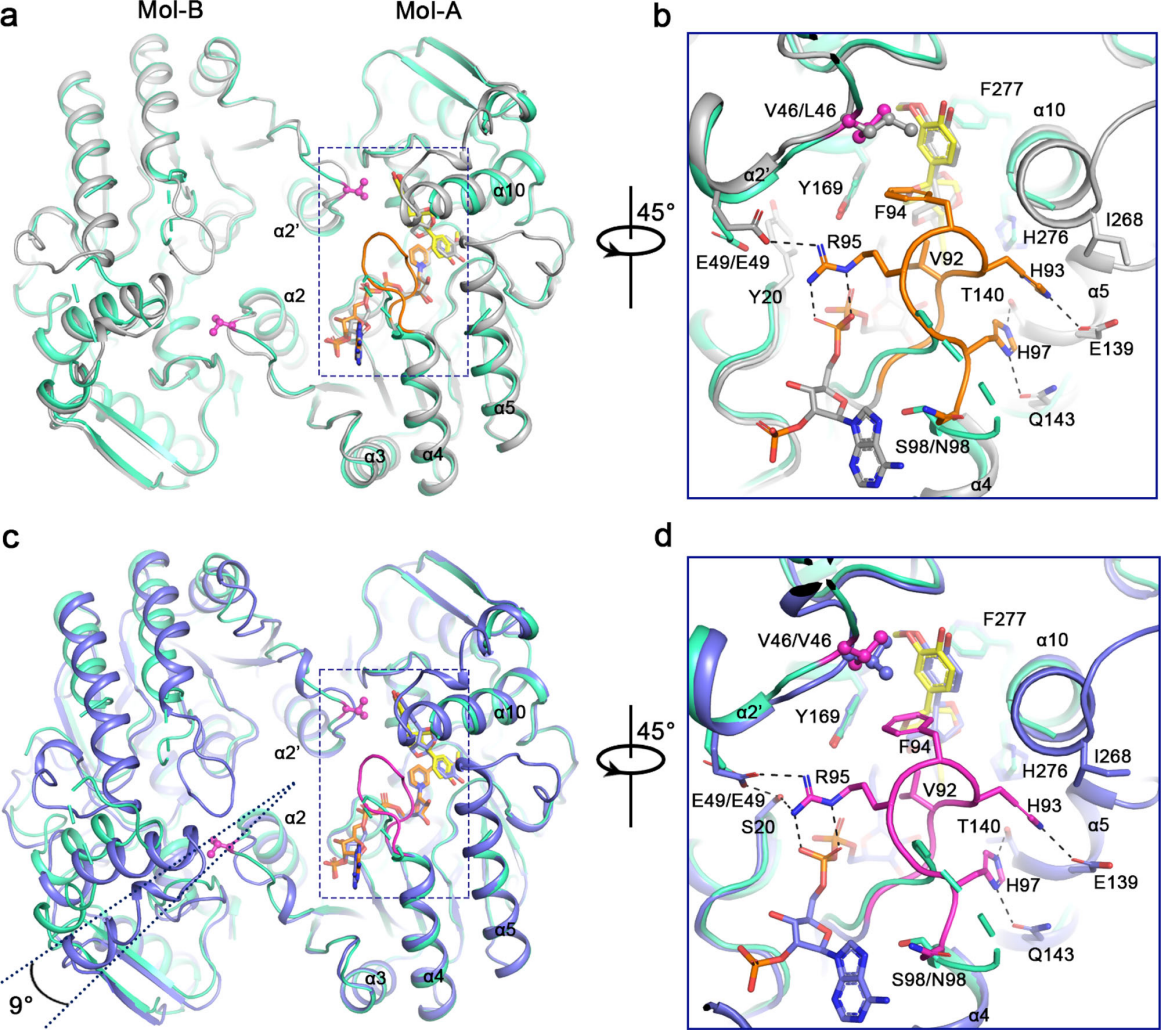
Structural differences among *Ii*PLR1, *At*PrR1, and *At*PrR2 indicating different catalytic capacities. **a** Structural alignment of dimerized *Ii*PLR1_NAP_ + PIN (green cyan) and *At*PrR1_NAP_ + PIN (light gray). **b** Zoom-in view of dashed box in **a**. The β4 loop of *At*PrR1_NAP_ + PIN is highlighted in orange. **c** Structural alignment of dimerized *Ii*PLR1_NAP_ + PIN (green cyan) and *At*PrR2_NAP_ + PIN (slate blue). **d** Zoom-in view of the dashed box in **c**. The β4 loop of *At*PrR2_NAP_ + PIN is highlighted in magenta. For brevity, NADP^+^ and some residues in *Ii*PLR1 are hidden. The cartoons are generated by PyMOL.

A previous study reported that the recombinant *At*PrR1 can only weakly reduce lariciresinol whereas *At*PrR2 lacks activity, which is in sharp contrast to all known PLRs^15^. To determine the mechanism underlying this difference in substrate specificity, we confirmed the relative lack of activity for *At*PrR1/2 (Fig. 1) and then carried out a structure analysis of *Ii*PLR1, *At*PrR1, and *At*PrR2. Each of *Ii*PLR1_NAP_ + PIN/*At*PrR1_NAP_ + PIN/*At*PrR2_NAP_ + PIN forms a homodimer, and superimposition of the protomers among the three complexes revealed RMSDs of 0.374, 0.365, and 0.308 Å, respectively (Fig. 3a, c and Supplementary Fig. 1). In contrast to *Ii*PLR1_NAP_ + PIN, the β4 loops of *At*PrR1_NAP_ + PIN and *At*PrR2_NAP_ + PIN can be clearly identified (Fig. 3 and Supplementary Fig. 4). These well-defined loops twist as an “8” shape and cover both the NADP^+^-binding and substrate-binding grooves. Within the twisted loop, His93 and His97 “grasp” helices α5 and α10, while Val92 and Phe94 interact directly with (+)-pinoresinol; Arg95 strongly interacts with NADP^+^ as well as each of the GXXGXXG motif and α2-helix from neighboring protomer of the dimer. Similar β4 loops are also observed in all other *At*PrR1 substrate/product-bound structures, whereas each β4 loop is disordered in the corresponding *Ii*PLR1 structures, which indicates that the β4 loop may participate in substrate selectivity and, hence, catalysis.

We further explored why the loops behaved differently between *Ii*PLR1 and the *At*PrRs. The amino acid sequences of the β4 loops in the three proteins are quite similar (Supplementary Fig. 5), but the residue corresponding to Ser98 at the C-terminal end of the loop in *Ii*PLR1 is replaced as Asn98 in *At*PrR1/2. Combining the sequence and structural data, the difference can be explained reasonably as follows: the serine side chain is short enough to remain beneath the guanine group of NADPH, whereas the asparagine side chain cannot do so owing to steric hindrance. Consequently, the asparagine lies nearly vertical to the guanine group and points upward in the structure shown in Fig. 3b, d, and thus the swing of the β4 loop is limited in the *At*PrR1_NAP structure. As substrate enters the catalytic site, β4 loop can fold and cover the substrate-binding groove (Fig. 3b, d and Supplementary Figs. 3b and 4a). Besides the β4 loop, Val46 in *Ii*PLR1 is replaced with Leu46 in *At*PrR1, which has the effect of compressing the substrate-binding pocket. Although Val46 is unchanged in *At*PrR2, the α2-helix and β2 loop from the neighboring protomer move closer to the substrate upon its entry at the catalytic site, further condensing the pocket. The relative movement of dimers between *At*PrR2 and *Ii*PLR1 (as suggested by the ~9° shift shown in Fig. 3c) could be induced by different dimer orientations. Two molecules of the dimer exhibit relative torsion in *At*PrR2, and consequently, Val46 is forced deeper into the substrate-binding pocket compared with what occurs in *Ii*PLR1. Therefore, the entrance and orientation of the substrate in *At*PrR1/2 is more tightly controlled than in *Ii*PLR1.

### Mutagenesis-based alteration of the substrate selectivity

**Fig. 4:**
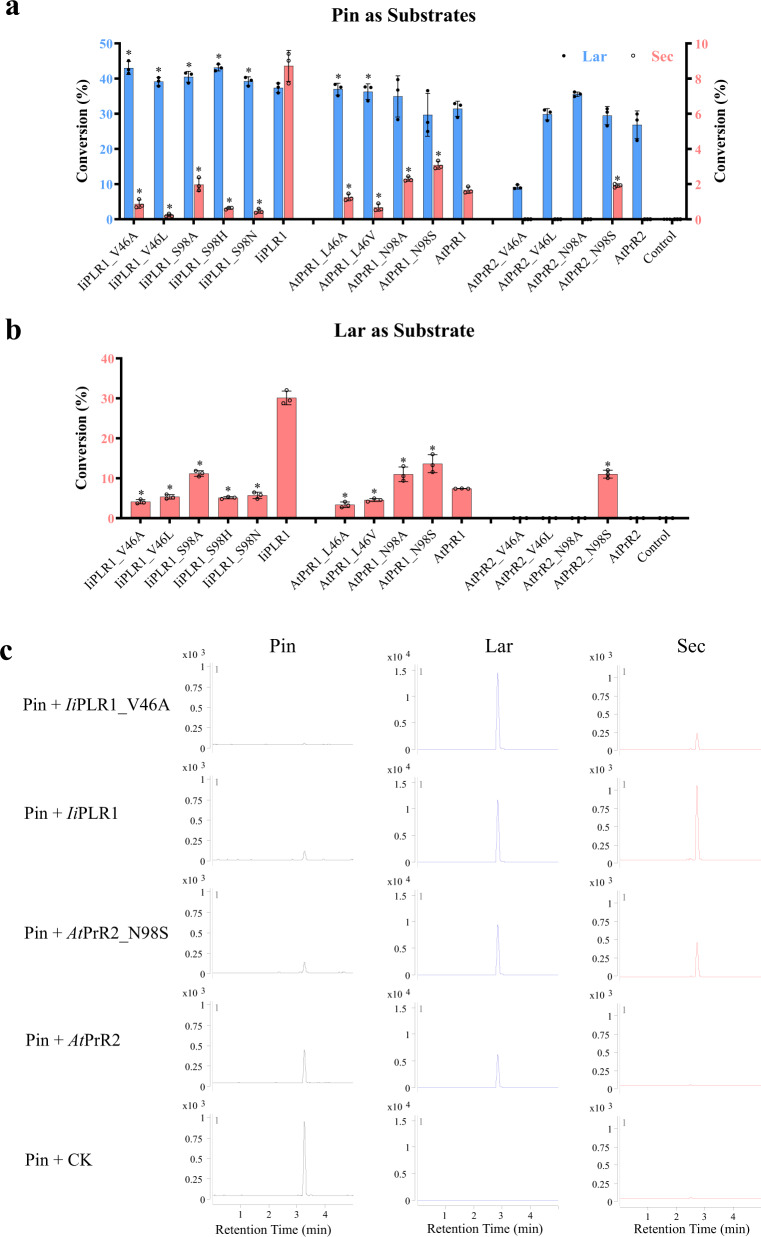
Percent conversion of pinoresinol to lariciresinol and subsequently to secoisolariciresinol by mutants of *Ii*PLR1, *At*PrR1 and *At*PrR2. **a** Conversion of pinoresinol into lariciresinol and subsequently to secoisolariciresinol. **b** Conversion of lariciresinol into secoisolariciresinol. Data are mean ± s.d. (*n* = 3 independent experiments). Asterisk (*) indicates significant difference from the wild-type enzyme (*P* < 0.05) analyzed by one-way ANOVA with Tukey’s multiple comparisons test. Source data underlying Fig. 4a, b are provided as a Source Data file. **c** LC-MS determination of the products as catalyzed by *Ii*PLR1, *Ii*PLR1_V46A, *At*PrR2 and *At*PrR2_N98S.

Based on the structural analysis of *Ii*PLR1, *At*PrR1 and *At*PrR2, the importance of the candidate amino acids controlling substrate specificity was verified through site-directed mutagenesis. Enzymatic assays using pinoresinol as substrate revealed that the *Ii*PLR1 mutations including V46A, V46L, S98A, S98H, and S98N somewhat enhanced the conversion rate of lariciresinol while significantly reduced that of secoisolariciresinol, and mutants V46A, S98A, and S98H had > 40% conversion rates for lariciresinol (Fig. 4a), suggesting that residues 46 and 98 are critical for the substrate preference. Taking V46A as an example for the kinetic analysis, its *K*_m_ value for pinoresinol (29.4 ± 1.62 μM) is comparable with that for lariciresinol (26.5 ± 0.60 μM), however its *V*_max_ for pinoresinol (3.22 ± 0.68 μM min^−1^) is 140-fold higher than that for lariciresinol (0.023 ± 0.0013 μM min^−1^), and its *k*_cat_*/K*_m_ for pinoresinol (3.88 ± 0.65 μM^−1^ min^−1^) is 126-fold higher than that for lariciresinol (0.031 ± 0.002 μM^−1^ min^−1^) (Table 2). Compared with wild-type *Ii*PLR1^18^, the activity of mutant V46A towards pinoresinol increases 4-fold, whereas that towards lariciresinol decreases 98% with regard to *k*_cat_*/K*_m_ values. These results indicate mutant V46A enhances catalytic efficiency for the first reaction but dramatically eliminates the second reaction. Consistent with the data for *Ii*PLR1, *At*PrR1 mutants L46A and L46V could enhance the conversion rate of lariciresinol and partially reduce that of secoisolariciresinol (Fig. 4a), which confirmed the importance of these two sites in substrate binding and product release thus in catalysis. As expected, mutants N98A and N98S in *At*PrR1 had increased activity for secoisolariciresinol production compared with wild type (Fig. 4a), strongly implying that residue 98 controls the swing of the β4 loop, which affects substrate binding and catalysis. Interestingly, *At*PrR2 mutant N98S could utilize lariciresinol to produce secoisolariciresinol, with a conversion rate of 1.91%, in contrast to the wild type which lacks this activity (Fig. 4a). Other *At*PrR2 mutants, including V46A, V46L, and N98A, varied in their activities for pinoresinol, as indicated by the relative rates of conversion to lariciresinol (Fig. 4a). Similar results were obtained for conversion of lariciresinol to secoisolariciresinol (Fig. 4b). Altogether, the structure-guided mutagenesis indeed could switch the substrate specificity of PLR/PrR, e.g., the *Ii*PLR1 mutant V46A had increased preference for pinoresinol but little catalytic activity for lariciresinol, whereas the *At*PrR2 mutant N98S gained the activity to catalyze the conversion of lariciresinol to secoisolariciresinol (Fig. 4c).

**Table 2:** Kinetic properties of *Ii*PLR1_V46A.

Substrate	*K*_m_ (μM)	*V*_max_ (μM/min)	*k*_cat_ (min^−1^)	*k*_cat_*/K*_m_ (μM^−1^ min^−1^)
(±)-pinoresinol	29.4 ± 1.62	3.22 ± 0.68	114.7 ± 24.1	3.88 ± 0.65
(±)-lariciresinol	26.5 ± 0.60	0.023 ± 0.0013	0.81 ± 0.045	0.031 ± 0.002

Data are expressed as mean ± s.d. with three independent experiments. Source data are provided as a Source Data file.

**Fig. 5:**
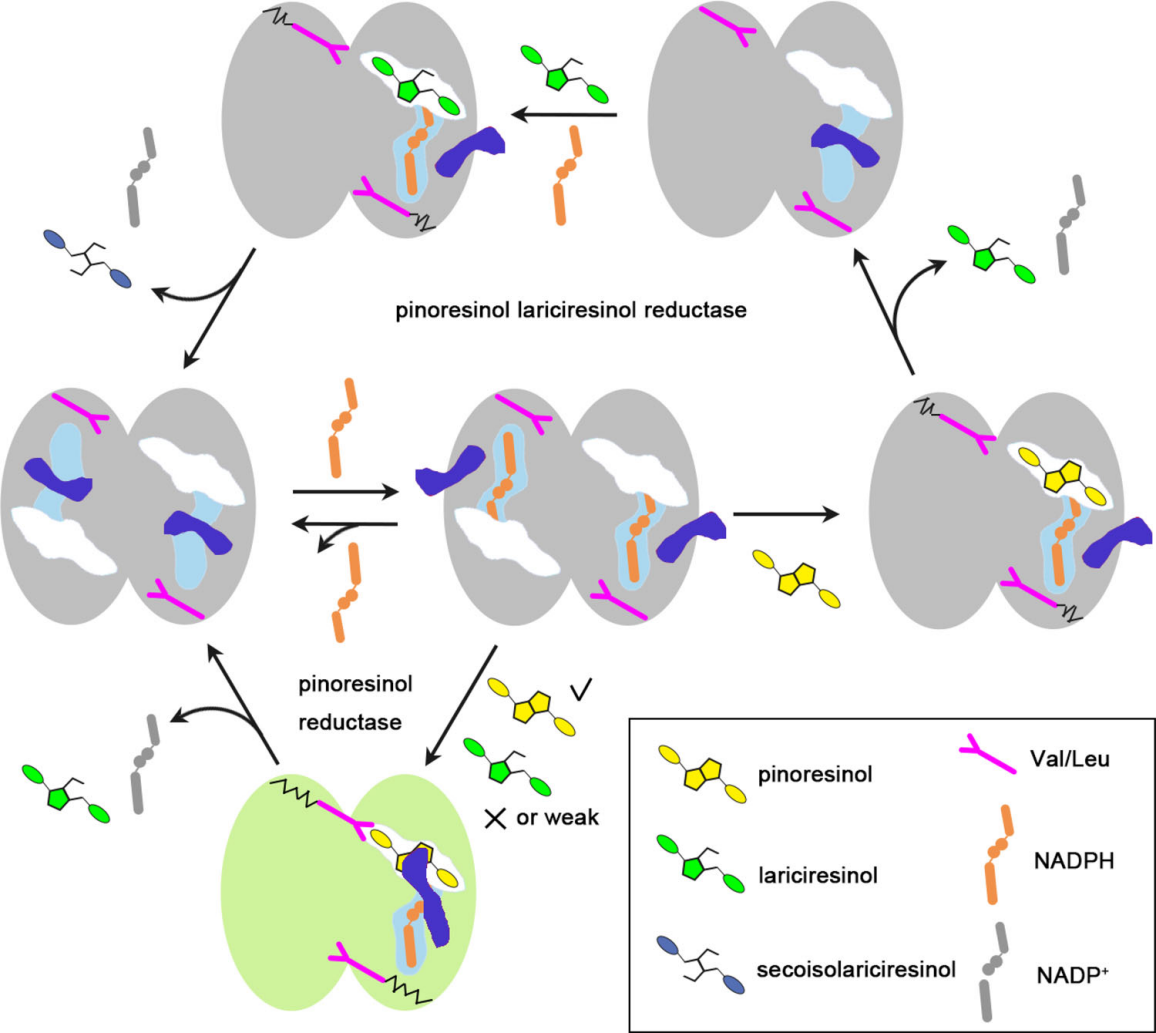
Model depicting the catalytic processes of PLRs and PrRs. The blue dumbbell-shaped objects represent switches composed of β4 loops. A movie showing how the enzymes change conformation throughout a single round of catalysis can be found in Supplementary Movie 1.

Taking structure and enzymology data together, we proposed a three-step catalytic mechanism for PLR based on its homodimerization. First, the protomers of dimeric PLRs recruit free NADPH through the very flexible β4 loop. Second, pinoresinol binds into one protomer via the substrate-binding groove, and the other protomer of the homodimer helps stabilize the substrate. Subsequently, pinoresinol receives H: from NADPH and be reduced to lariciresinol released later. Third, free lariciresinol is bound by another reductive PLR molecule and fixed by another homodimer, and then the lariciresinol is reduced to secoisolariciresinol and finally released (Fig. 5 and Supplementary Movie 1).

Importantly, the PrRs have more strict requirements for the binding and orientation of lariciresinol compared with PLRs, so PrRs cannot efficiently carry out the third step (Fig. 5). Hence, the substrate-specificity mechanism of PLRs/PrRs could be that residues located around the substrate-binding pocket and within the loop, together with residues that promote homodimerization, form the appropriate hydrophobic environment for binding a specific substrate.

### Mutation increases lariciresinol and reduces secoisolariciresinol production in vivo

Enzymatic assays indicated that certain *Ii*PLR1 mutants had increased activity for producing lariciresinol from pinoresinol in vitro (Fig. 4). Therefore, these *Ii*PLR1 mutant genes were selected for lariciresinol production using pinoresinol-producing *E. coli*^19^. Because matairesinol, which is derived from secoisolariciresinol, is detectable only when CueO (multicopper oxidase), PLR and SDH (secoisolariciresinol dehydrogenase) are individually expressed in cells^20^, each of wild-type *Ii*PLR1 and its mutants were co-cultured in pinoresinol-producing *E. coli*^19^. Consistent with the enzyme assay results, *Ii*PLR1_V46A produced the greatest amount of lariciresinol (997.79 mg L^−1^ compared with 936.14 mg L^−1^ for wild-type). However, mutants V46L, S98A, S98H, and S98N were not as efficient as wild-type cells at producing lariciresinol, which was opposite to the results from in vitro enzyme assays. This may reflect the potential effects of complex metabolic networks and feedback mechanisms in vivo, which are not relevant to in vitro enzyme assays. Moreover, the provision of NADPH is tightly regulated in prokaryotic systems, which also may influence the activity of PLRs.

**Fig. 6:**
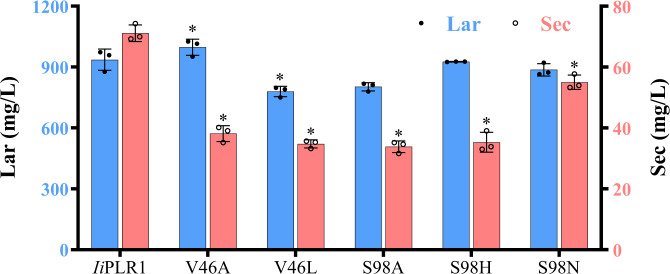
Lariciresinol production through co-culture of different strains harboring a plasmid encoding *Ii*PLR1 or its single-site mutants with pinoresinol-producing cells. Data are mean±s.d. (*n* = 3 independent experiments). Asterisk (*) indicates significant difference from the wild-type enzyme (*P* < 0.05) analyzed by one-way ANOVA with Tukey’s multiple comparisons test. Source data are provided as a Source Data file.

Notably, all the *Ii*PLR mutants produced significantly less secoisolariciresinol than wild-type cells, i.e., by 22.7–52.5%; in particular, *Ii*PLR_V46A produced 46.4% less secoisolariciresinol than wild type (Fig. 6). These results paralleled those obtained in vitro with the *Ii*PLR1 mutants in which there was elimination of the second catalytic step, i.e., the conversion of lariciresinol to secoisolariciresinol (Fig. 4). Taken together, our results establish a promising route for the production of lariciresinol by synthetic biology strategies, and mutant *Ii*PLR_V46A mutant would be a good candidate for use in the large-scale production of the pharmaceutically valuable compound lariciresinol.

## Discussion

The molecular mechanism of substrate selectivity of PLR/PrR has attracted particular interest owing to the key role of these enzymes in lignan biosynthesis. However, the lack of structural results—especially for PLRs/PrRs in complex with different substrates—has limited our understanding of the mechanism underlying enzyme specificity. In the present study, we characterized crystal structures of *Ii*PLR1, *At*PrR1, and *At*PrR2 in complex with their various substrates. Several residues participating in substrate binding and catalysis were identified either directly or indirectly based on structural analysis, and these residues were validated by enzyme assays. All these data provide solid evidence to explore the mechanistic basis of substrate selectivity for PLRs/PrRs. Besides residues 46 and 98 in *Ii*PLR1 and *At*PrRs that we identified as being critical for binding and catalysis, residues Phe166, Tyr169, Phe170, His276, and Phe277 within the substrate-binding groove were also strongly correlated with enhanced substrate binding and catalysis. We further deduced that any residue in PLRs/PrRs around the hydrophobic groove or affecting homodimerization may impact the conformation of the active site, thereby dictating substrate selectivity (Supplementary Figs. 5 and 6). Consequently, it is not difficult to understand why mutant L174I of *C. sinensis* PLR1 can hardly reduce pinoresinol and specifically catalyze the conversion of lariciresinol to secoisolariciresinol^16^, i.e., because Leu174 points directly toward Tyr163 and thus may indirectly promote substrate recognition.

In addition, PLRs also display substrate stereochemical selectivity, which contributes to the enantiomeric diversity of lignans^21–24^. We found this to be true for *Ii*PLR1, which gave comparable *k*_cat_/*K*_m_ values for both (±)-pinoresinol and (±)-lariciresinol in the range of 0.9–1.6 µM^−1^ min^−1^, although no experiments with respect to the enantio-specificity of this enzyme have been performed^12,18^. Despite past research on this topic, however, the mechanism underlying the substrate stereochemical selectivity of PLRs remains unclear. Comparison of the enantiospecifically opposite PLRs *Tp*PLR1 and *Tp*PLR2 suggests that F164, V268, and L272 in *Tp*PLR1 contribute to the catalysis of (−)-pinoresinol, whereas L164, G268, and F272 in *Tp*PLR2 prefer to bind (+)-pinoresinol^17^. Nevertheless, site-directed mutagenesis carried out in flax indicates that these positions are insufficient to determine enantiospecificity^21^. Based on amino-acid sequence and structural analyses, it seems that residues Phe94 and Phe277 in *Ii*PLR1/*At*PrR1 may act in concert to determine the enantiospecificity of PLRs (Supplementary Fig. 6). These two residues are highly conserved in PLRs that have no enantio-specificity, whereas Ile94 and Tyr277 are present in PLRs that have a strict substrate preference for (+)-pinoresinol. Moreover, in *Linum usitatissimum* PLR1 (*Lu*PLR1), which has strict enantiospecificity for (–)-pinoresinol, a leucine residue is deleted as are two other residues on β4 loop (corresponding to the β4 loop of *Ii*PLR1, where Phe94 is located). Unfortunately, we could not obtain sufficient amounts of the enantiomerically pure substrates to carry out the experiments necessary to establish the enantio-selectivity.

Nature uses a dazzling array of enzymes to produce diverse natural products. However, some modifications are challenging to control because the relative lack of substrate specificity often generates undesired byproducts. *Ii*PLR1 plays an important role in the biotechnological production of lariciresinol^18^, which represents the most important component for the antibacterial, antiviral, and the immune-regulatory effects of the traditional Chinese medicine Radix Isatidis^3,25,26^. The fact that *Ii*PLR1 can efficiently utilize both pinoresinol and lariciresinol as substrates^18^ suggests that the biosynthetic efficiency towards the pharmaceutically valuable compound lariciresinol in Radix Isatidis has been hampered by the relatively low substrate specificity of *Ii*PLR1. In our present work, structure-guided mutagenesis successfully switched the substrate specificity of *Ii*PLR1, leading to overproduction of lariciresinol and reduced production of secoisolariciresinol by *E. coli*. Our study provides insight into the molecular mechanism underlying the substrate specificity of PLRs/PrRs, and paves the way for the manufacture of lariciresinol through microbial fermentation. Moreover, this work suggests the possibility of using targeted mutagenesis of *Ii*PLR to improve the efficiency of synthesizing bioactive compounds in *I. indigotica* using gene-editing technologies^27,28^.

## Methods

### Phylogenic analysis of plant PLRs

Phylogenetic relationships were analyzed using the maximum likelihood method with the pairwise deletion option in MEGA 6.06. Tree reliability was estimated using a bootstrap analysis of 1000 replicates^29^. Plant PLR amino-acid sequences used in the phylogenic analysis were retrieved from GenBank, including *Tp*PLR1 (AAF63507.1), *Tp*PLR2 (AAF63508.1), *Tp*PLR3 (AAF63509.1), *Tp*PLR4 (AAF63510.1), *Pp*PLR (AHL21381.1), *La*PLR1 (CAH60857.1), *Lu*PLR1 (CAH60858.1), *Lu*PLR2 (ABW24501.1), *Lp*PLR1 (ABM68630.1), *Lc*PLR1 (ABW86959.1), *Ph*PLR (ACF71492.1), *Tc*PLR1 (AZL88516.1), *Tc*PLR2 (AZL88517.1), *Fi*PLR1 (AAC49608.1), *Ii*PLR1(AEA42007.1), *At*PrR1 (NP_174490.1), and *At*PrR2 (NP_193102.1).

### Heterologous expression of *Ii*PLR1, *At*PrR1, and *At*PrR2 in *E. coli*

Total RNA was extracted from leaves of wild-type *I. indigotica* or *A. thaliana* using TRIzol Reagent (GIBCO BRL). The mRNA was reverse transcribed with oligo dT to generate cDNA as a template for PCR. Full-length cDNA sequences of *Ii*PLR1 (GenBank accession no. JF264893), *At*PrR1 (AY065214) and *At*PrR2 (BT002882) were cloned into pET-duet-1 (Novagen, USA) to generate *Ii*PLR1-pET, *At*PrR1-pET, and *At*PrR2-pET, respectively. The primers used are listed in Supplementary Table 1. *E. coli* Rosetta (DE3) cells were transformed with purified plasmid DNA and then grown at 37 °C to an OD_600_ of 0.8. Then, protein expression was induced by adding 0.5 mM isopropyl β-d-thiogalactoside (IPTG, final concentration) with incubation overnight at 16 °C. Cells were collected, resuspended in buffer A (20 mM Tris-HCl pH 8.0, 100 mM NaCl), and lysed with a French press. The lysate was centrifuged at 20,000×*g* for 45 min, and the supernatant was applied to a Ni-NTA column equilibrated with buffer A supplemented with 25 mM imidazole. Bound protein was eluted using buffer A containing 250 mM imidazole and was concentrated for further purification on a Superdex-200 column equilibrated with buffer A. Protein purity was assessed by SDS-PAGE (12% polyacrylamide), and protein concentration was determined by the Bradford method^30^.

### Crystallization, data collection, and structure determination

The full-length *Ii*PLR1/*At*PrR1/*At*PrR2 were purified as described above and concentrated to 5–10 mg mL^−1^ for crystallization. Aliquots of each concentrated protein sample were mixed 1:1 with reservoir solution, and crystals were grown at 20 or 4 °C in one week using the sitting-drop vapor-diffusion method. For co-crystals, protein was combined with NADP^+^ at a 1:5 molar ratio, and protein with NADP^+^ and substrate/product at a 1:5:10 molar ratio. For reservoir solutions, *Ii*PLR1 apo and co-crystals were grown with 0.2 M sodium citrate tribasic, 0.1 M sodium citrate/citric acid, pH 4.0 and 20% polyethylene glycol (PEG) 3350; *At*PrR1 apo crystals were grown in 0.2 M lithium chloride, 20% w/v PEG 3350; *At*PrR1_NAP, *At*PrR1_NAP_ + PIN, *At*PrR1_NAP_ + LAR and *At*PrR1_NAP_-SEC were grown in 0.2 M sodium fluoride, 20% w/v PEG 3350; *At*PrR1_NAP_-PIN and *At*PrR1_NAP_-LAR crystals were grown in 0.2 M sodium malonate, pH 6.0, 20% w/v PEG 3350; *At*PrR2 apo crystals were grown in 0.2 M magnesium chloride, 0.1 M sodium HEPES, pH 7.5 and 25% PEG 3350; *At*PrR2_NAP_ + PIN crystals were grown in 2.1 M DL malic acid, pH 7.0. The crystals were cryoprotected by serial transfers into reservoir solutions supplemented with 30% (v/v) glycerol and then flash-cooled in liquid nitrogen. Data collections were performed at the BL17U1 and BL19U1 beamline of the Shanghai Synchrotron Radiation Facility. The data were processed with HKL3000^31^, and the initial phase was determined by molecular replacement with Phenix^32^ using the crystal structure of *Tp*PLR1 (PDB ID: 1QYD [10.2210/pdb1qyd/pdb]) as a template. The structure models were firstly auto-built in Coot^33^ and then refined by iterative rounds of manual adjustment with Coot and refinement with Phenix. The statistics of data collection and structure refinement are shown in Supplementary Tables 2–5.

### Site-directed mutagenesis of *Ii*PLR1, *At*PrR1, and *At*PrR2 and enzymatic assays

Single-site mutagenesis was achieved through one-step PCR, and mutants were verified with Sanger sequencing. All primers are listed in Supplementary Table 1. After expression and purification of recombinant enzymes under the aforementioned conditions, the results for the enzyme assays for mutants were compared with those for wild-type recombinant *Ii*PLR1, *At*PrR1, and *At*PrR2 as follows.

Enzyme activity assays were conducted strictly according to our previous work^18^. Assay mixtures (1 mL) consisted of TG buffer (50 mM Tris-HCl, 10% [w/v] glycerol, pH 7.0), 150 μM NADPH], 100 μM pinoresinol, or 100 μM lariciresinol and 5 μg of purified protein. Assays without a fusion protein were used as controls. Protein, buffer, and substrate were pre-incubated for 5 min at 30 °C, and each reaction was initiated by addition of NADPH and terminated after 30 min by addition of 300 μL ethyl acetate. Each assay mixture was extracted with ethyl acetate (3 × 300 μL total). The combined ethyl acetate phases were dried under vacuum, and the residue was dissolved in 1 mL methanol. Conversion rate was then determined. The content of pinoresinol, lariciresinol and secoisolariciresinol was determined by LC-MS using a triple-quadrupole mass spectrometer (Model 6410, Agilent, Santa Clara, CA) following our published methods^18^. MassHunter Qualitative Analysis B.06.00 was used for the data analysis. The selected transitions of *m*/*z* were 357 → 151 for pinoresinol, 359 → 329 for lariciresinol, and 361 → 164 for secoisolariciresinol. All standards were purchased from Sigma-Aldrich (St. Louis, MO).

For determination of *V*_max_ and *K*_m_ values for *Ii*PLR1_V46A, 10 different concentrations of substrate (pinoresinol or lariciresinol; 5–200 μM) and 1 μg purified protein were used. Samples were incubated at 30 °C for 5 min (during which substrate consumption was ≤10%). Samples without protein were used as controls. The rate of substrate consumption was calculated for kinetic analysis. *V*_max_ and *K*_m_ values were determined from Lineweaver-Burk plots, and *k*_cat_ was determined by dividing *V*_max_ by the enzyme concentration.

### Bioconversion

For the production of lariciresinol, biotransformation was divided into two modules, namely the accumulation and conversion of the precursor, pinoresinol. *E. coli* strain strOpr2 carrying plasmid pET28a-Prx02-PsVAO was used to produce pinoresinol^19^, whereas *E. coli* BL21(DE3) carrying a plasmid encoding *Ii*PLR1 or its mutants was used for conversion of pinoresinol to lariciresinol. These *E. coli* strains were cultured in LB medium at 37 °C with shaking (220 rpm) for 12 h as seed cultures, and then a 2% seed culture was transferred to a 250-mL shaker flask containing 25 mL TB medium. After culturing for 2–2.5 h at 37 °C and 220 rpm, 500 μM IPTG (final concentration) was added to the medium with continued cultivation for 12 h at 25 °C and 220 rpm. These cells were used for pinoresionol accumulation and conversion, respectively. Cells from *E. coli* strain strOpr2 were harvested by centrifugation at 4 °C and 3724×*g* for 30 min and then resuspended in phosphate-buffered saline (pH 7.0) to adjust the OD_600_ value to 20. Then 0.15% (v/v) eugenol was added into 15 mL of the resuspension at 0, 1, 3, 5, and 7 h for pinoresinol accumulation (20 °C, 220 rpm). At 9 h, 15 ml of a culture of *E. coli* expressing *Ii*PLR1 and each mutant (OD_600_ = 20) was added to determine the conversion of pinoresinol to lariciresinol (25 °C, 220 rpm), and samples were taken after 20 h. The concentration of each of lariciresinol and secoisolariciresinol was determined by HPLC.

### Statistical analysis

All the experiments in this paper were repeated at least three times and results from representative data sets are presented. GraphPad Prism (version 9.1.0) was used for the statistical analysis. The statistical evaluations used one-way analysis of variance (ANOVA) with multiple comparisons, followed by Tukey tests. The results were considered statistically significant at *P < 0.05.

## Reporting summary

Further information on research design is available in the Nature Research Reporting Summary linked to this article.

## Supplementary information

Supplementary Information

Peer Review File

Supplementary Movie 1

Description of additional supplementary files

Reporting Summary

## Data Availability

Data supporting the findings of this work are available within the paper and its Supplementary Information files. The atomic coordinates and structure factors for the structures have been deposited in the Protein Data Bank with accession codes 7CS2 (Apo structure of dimeric *Ii*PLR1), 7CS3 (*Ii*PLR1 with NADP^+^), 7CS4 (*Ii*PLR1 with NADP^+^ and (+)pinoresinol), 7CS5 (*Ii*PLR1 with NADP^+^ and (−)pinoresinol), 7CS6 (*Ii*PLR1 with NADP^+^ and (−)lariciresinol), 7CS7 (*Ii*PLR1 with NADP^+^ and (+)secoisolariciresinol), 7CS8 (*Ii*PLR1 with NADP^+^ and (−)secoisolariciresinol), 7CS9 (*At*PrR1 in apo form), 7CSA (*At*PrR1 with NADP^+^), 7CSB (*At*PrR1 with NADP^+^ and (+)pinoresinol), 7CSC (*At*PrR1 with NADP^+^ and (−)pinoresinol), 7CSD (*At*PrR1 with NADP^+^ and (+)lariciresinol), 7CSE (*At*PrR1 with NADP^+^ and (−)lariciresinol), 7CSF (*At*PrR1 with NADP^+^ and (−)secoisolariciresinol), 7CSG (*At*PrR2 in apo form), 7CSH (*At*PrR2 with NADP^+^ and (+)pinoresinol). The initial phase was determined by molecular replacement using the crystal structure of *Tp*PLR1 (PDB ID: 1QYD [10.2210/pdb1qyd/pdb]) as a template. The source data underlying Figs. 2g, 4a, b, and 6, as well as Table 2 are provided as a Source Data file. All data generated and analyzed during the current study are available from the corresponding authors upon reasonable request. A reporting summary for this Article is available as a Supplementary Information file. Source data are provided with this paper.

## Source data

Source Data
