# Supplementary material for: Structure-based engineering of substrate specificity for pinoresinol-lariciresinol reductases
Source: Nat Commun. 2021 May 14;12:2828. doi: 10.1038/s41467-021-23095-y (PMC8121951; doi:10.1038/s41467-021-23095-y)
Supplement: Supplementary file 1 — Supplementary Information [file 41467_2021_23095_MOESM1_ESM.pdf]

Supplementary Information

Structure-based engineering of substrate specificity for pinoresinol-lariciresinol reductases

Ying Xiao, Kai Shao, Jingwen Zhou et al.

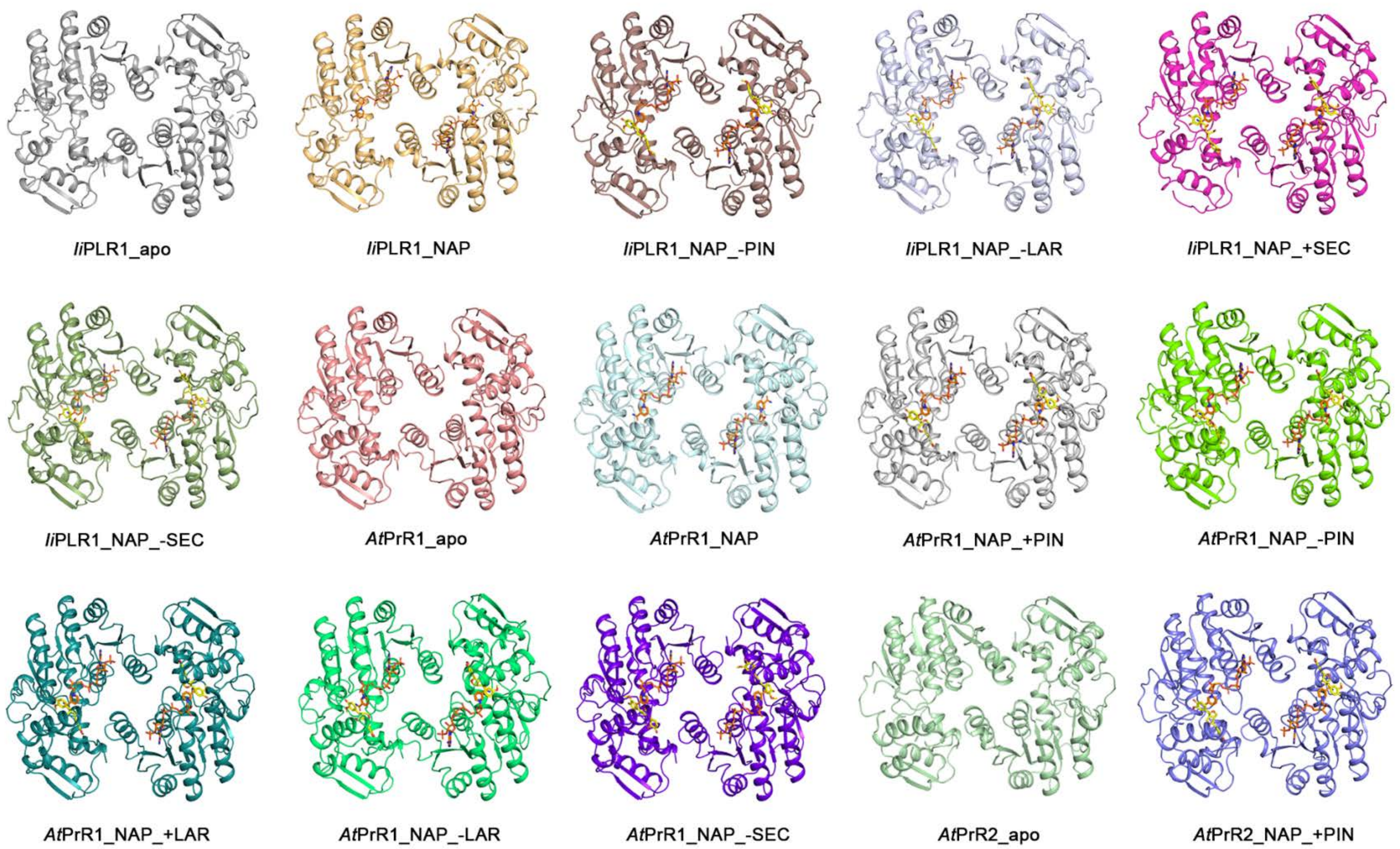

Supplementary Fig. 1. Dimer organization of the resolved *Ii*PLR/*At*PrR structures.

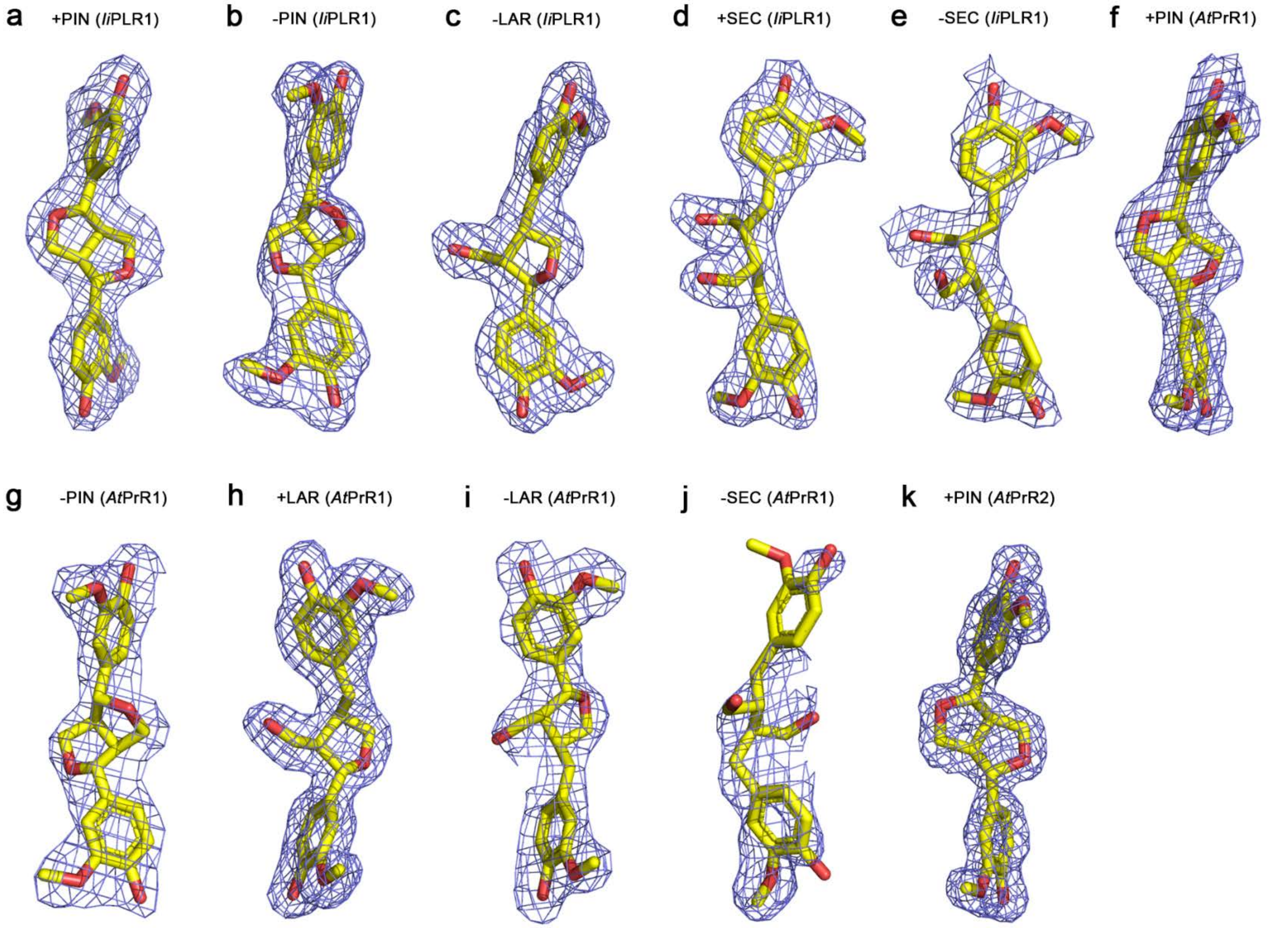

Supplementary Fig. 2. Electron density maps of substrate/product bound in the relevant structures. a-e, Electron density maps of +PIN, -PIN, -LAR, +SEC and -SEC in *Ii*PLR1 are shown, respectively. **f-j**, Electron density maps of +PIN, -PIN, +LAR, -LAR and -SEC in *At*PrR1 are shown, respectively. **k**, Electron density map of +PIN in *At*PrR2. Superimposed on these coordinates are omit maps (2Fo-Fc, contoured at 1.0 σ).

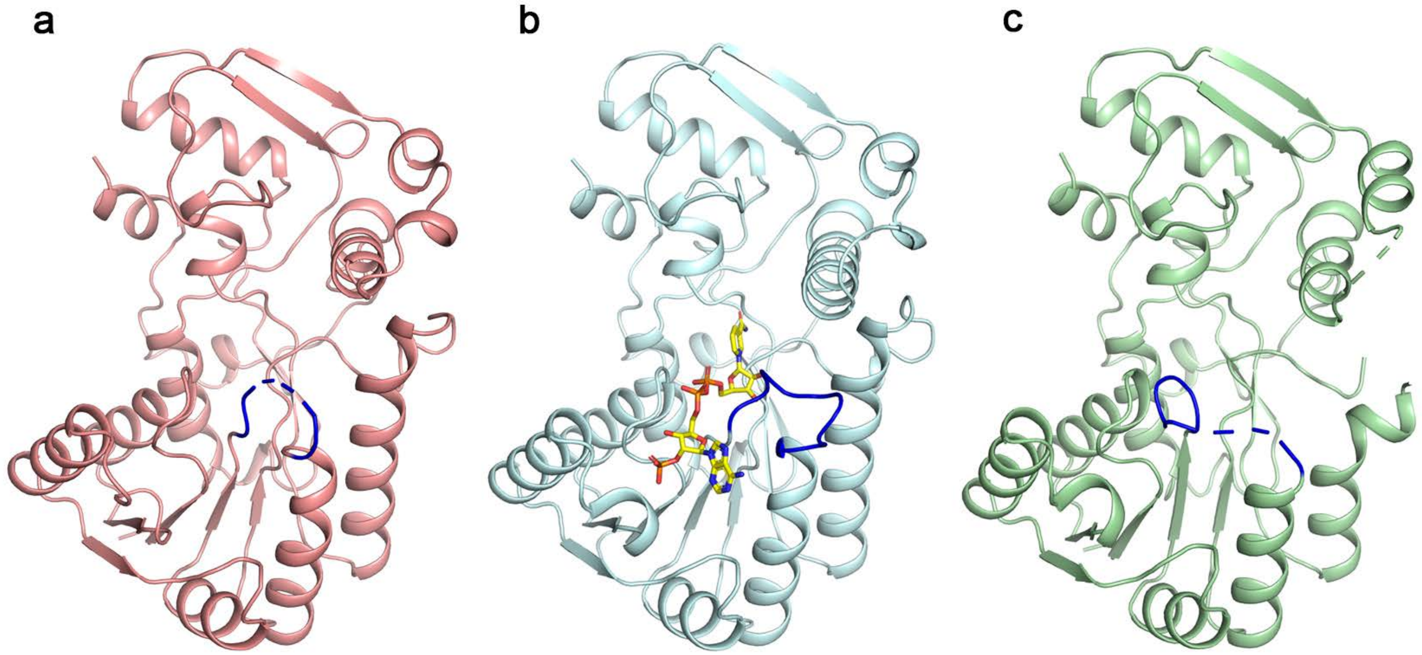

Supplementary Fig. 3. Structures of β4 loops in *At*PrR1/*At*PrR2. a-c, β4 loops (marine) of *At*PrR1_apo, *At*PrR1_NAP and *At*PrR2_apo are shown as light pink, pale cyan and light green, respectively.

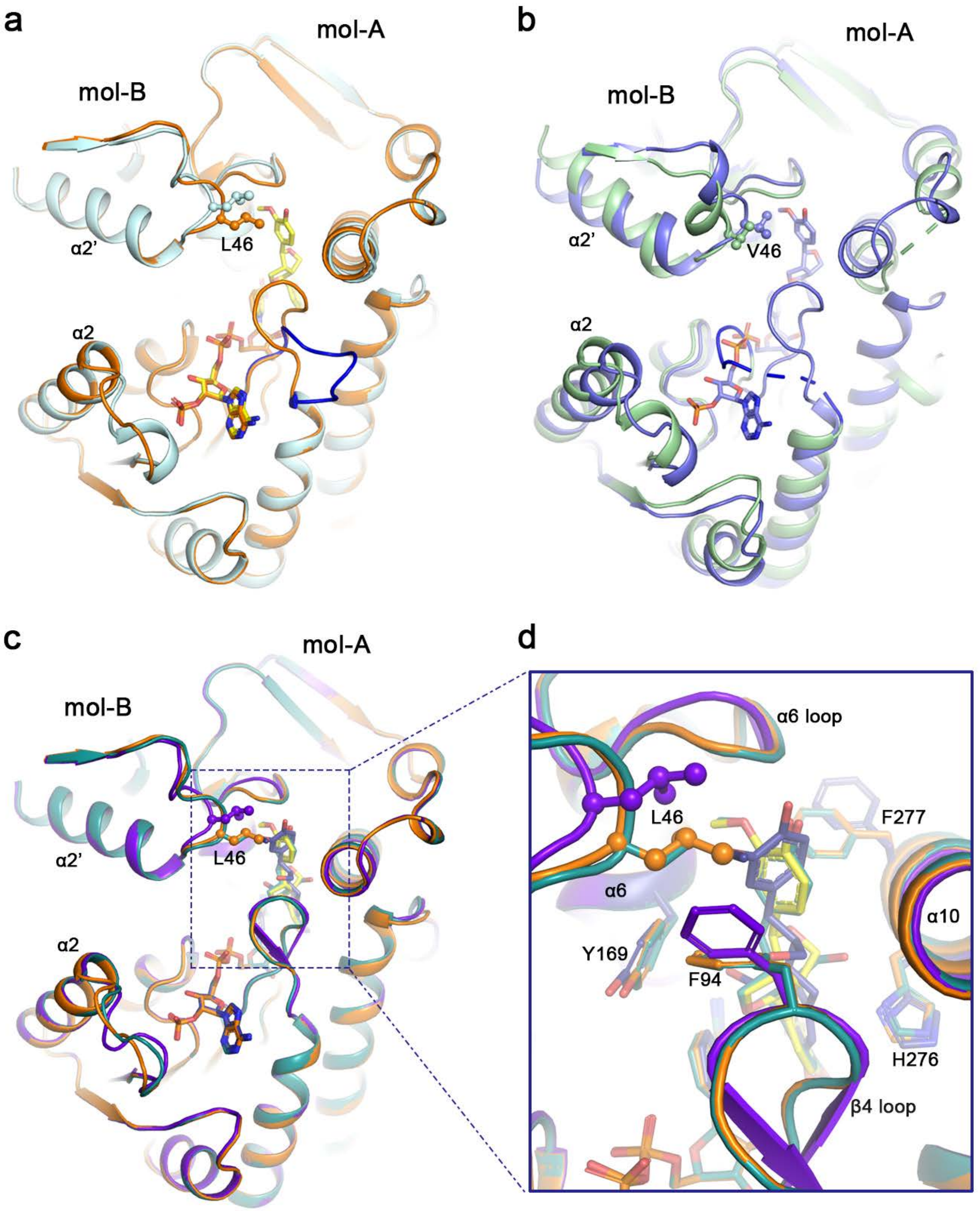

Supplementary Fig. 4. Structure comparison indicating the catalytic mechanism of *At*PrR1/*At*PrR2. a, Structural superposition of *At*PrR1_NAP (pale cyan) and *At*PrR1_NAP_+PIN (orange). **b,** Structural superposition of *At*PrR2_apo (light green) and *At*PrR2_NAP_+PIN (slate). **c,** Structural superposition of *At*PrR1_NAP_+PIN, *At*PrR1_NAP_+LAR (deep teal), and *At*PrR1_NAP_-SEC (purple blue). **d,** Room-in view of the substrate/product-binding groove. The (+)-pinoresinol, (+)-lariciresinol and (-)-secoisolariciresinol are shown as sticks colored yellow, deep teal and purple blue, respectively.

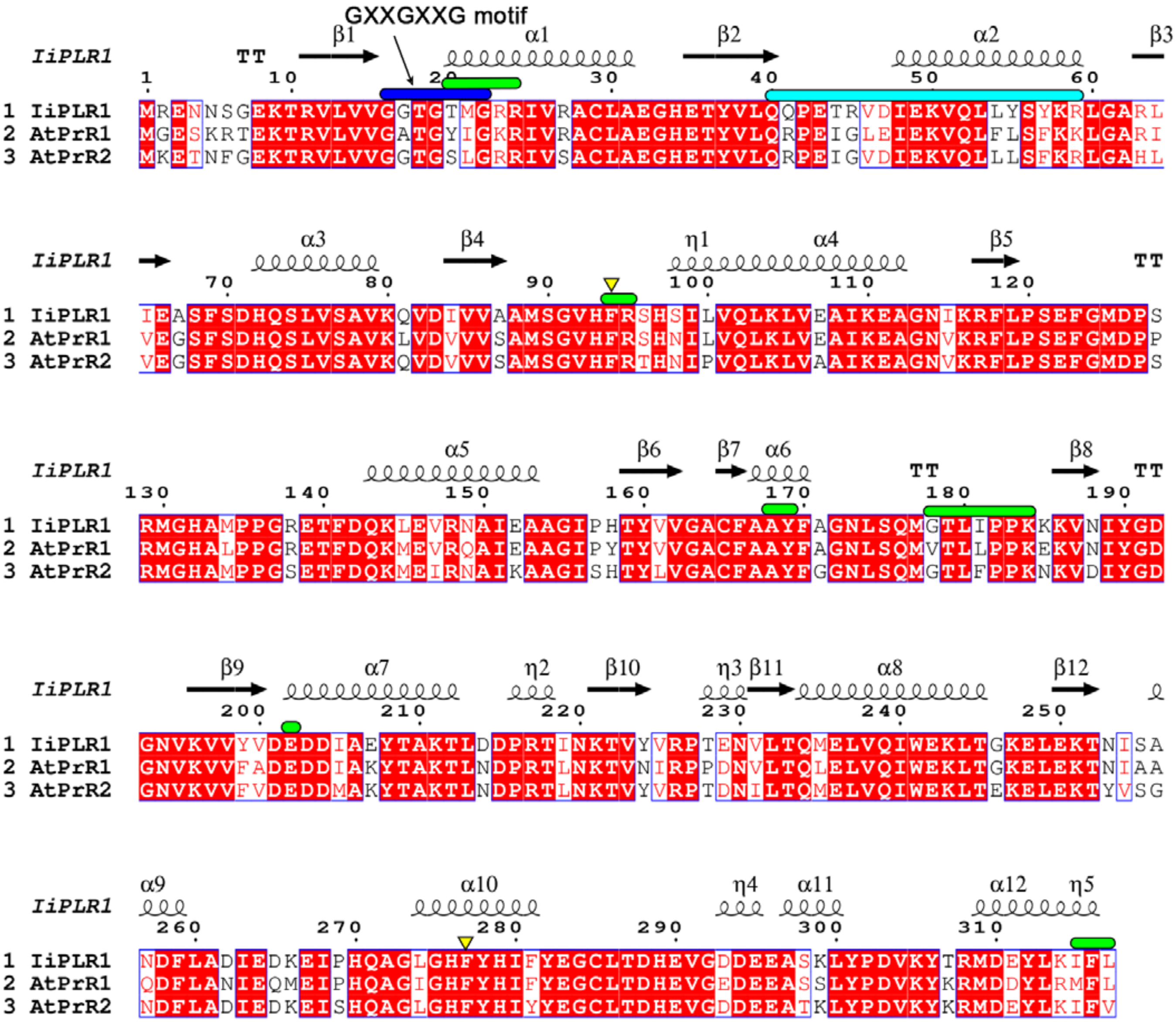

Supplementary Fig. 5. Amino-acid sequence alignment of *Ii*PLR and *At*PrRs. The secondary structure elements of *Ii*PLR1 are placed on top. The GXXGXXG motif is indicated with blue solid lines. Regions constituting dimer interfaces of *At*PrRs from Mol-A and Mol-B are indicated with green and cyan solid lines, respectively. Residues that may participate in chiral selection are indicated with yellow triangles. Species are: Ii, *Isatis indigotica*; At, *Arabidopsis thaliana*.

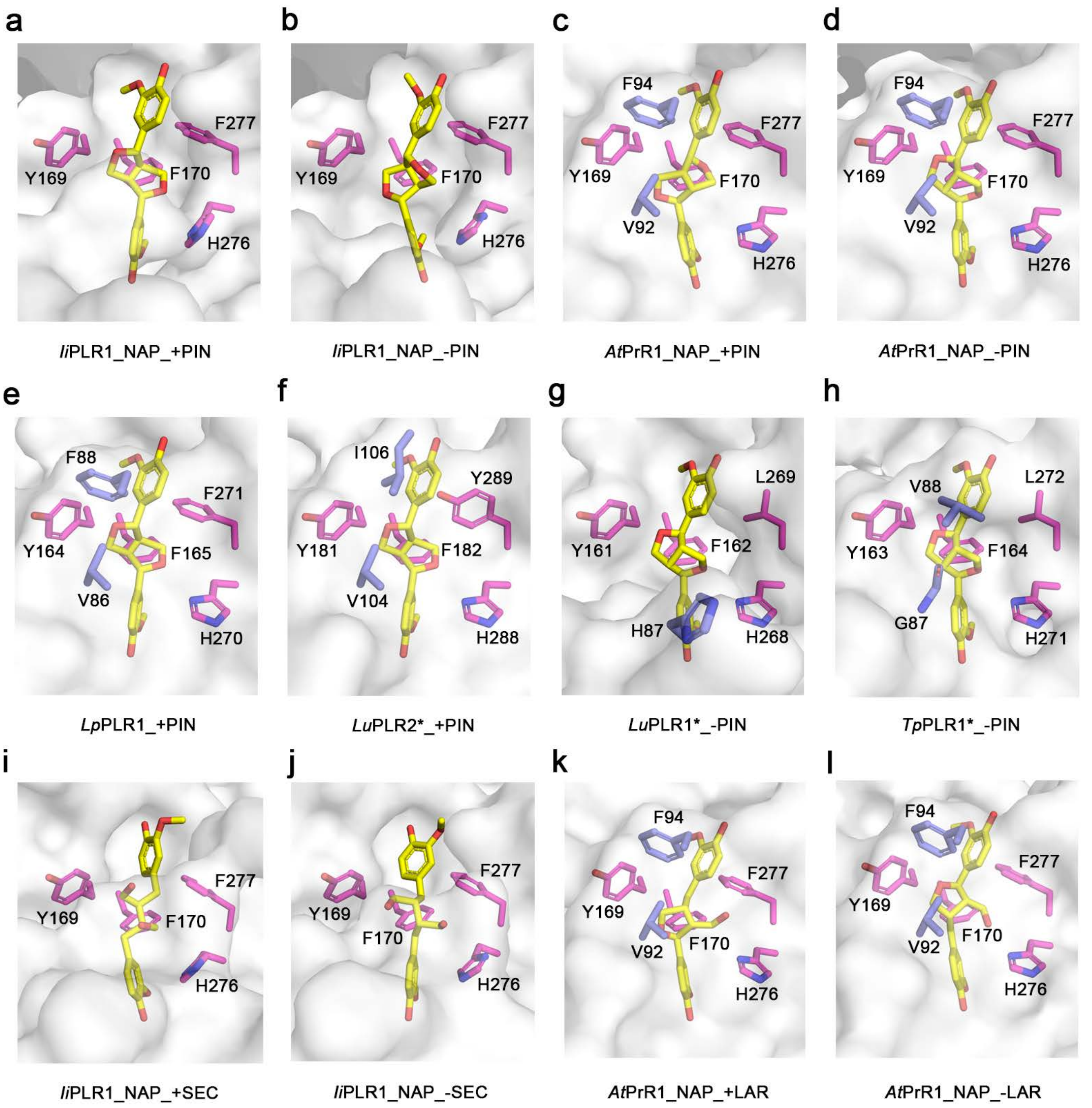

Supplementary Fig. 6. Comparison of chiral substrates and their surrounding amino acids. a–d, Structures with (+) or (–)-pinoresinol bound are shown as surface models. **e–h,** Modeled structures with aligned (+) or (–)-pinoresinol using swiss-model. **i–l,** Structures with (+) or (–)-lariciresinol/secoisolariciresinol bound are shown as a surface model. Species are: Lp, *Linum perenne*; Lu, *Linum usitatissimum*; Tp, *Thuja plicata*. *PLR displays strict selectivity for a particular enantiomeric substrate.

Supplementary Table 1. Primers using for cloning wild type and generating mutations

WT/Mutations	Primer	Sequence (5'→3')
*Ii*PLR1	*Ii*PLR1 F	T CAC CAT TCT GGATCC atgagagagaataat
	*Ii*PLR1 R	GCTCGAATTCGGATCC ctagaggaatatttt
*At*PrR1	*At*PrR1 F	T CAC CAT TCT GGATCC ATGGGAGAGA GCAAA
	*At*PrR1 R	GCTCGAATTCGGATCC TCAGAGGAACATTCT
*At*PrR2	*At*PrR2 F	T CAC CAT TCT GGATCC ATGAAAGAGA CTAAT
	*At*PrR2 R	GCTCGAATTCGGATCC TTATACGAAAATTTT
*Ii*PLR1_S98A	S98A F	Cactttcgcagccac GCA atccttgttcagctc
	S98A R	gagctgaacaaggat TGC gtggctgcgaaagtG
*Ii*PLR1_S98N	S98N F	Cactttcgcagccac AAT atccttgttcagctc
	S98N R	gagctgaacaaggat ATT gtggctgcgaaagtG
*Ii*PLR1_S98H	S98H F	Cactttcgcagccac CAT atccttgttcagctc
	S98H R	gagctgaacaaggat ATG gtggctgcgaaagtG
*Ii*PLR1_V46A	V46A F	cagccggagaccaga GCA gacatcgagaaggtg
	V46A R	caccttctcgatgtc TGC tctggtctccggctg
*Ii*PLR1_V46L	V46L F	cagccggagaccaga CTG gacatcgagaaggtg
	V46L R	caccttctcgatgtc CAG tctggtctccggctg
*Ii*PLR1_I181F	I181F F	caaatgggaactttg TTT cctccaaagaaaaaa
	I181F R	ttttttctttggagg AAA caaagttcccatttg
*Ii*PLR1_ I181A	I181A F	caaatgggaactttg GCA cctccaaagaaaaaa
	I181A R	ttttttctttggagg TGC caaagttcccatttg
*At*PrR1_N98A	N98A F	CACT TCCGTAGCCA T GCA ATTCTAGTCCAGCTC
	N98A R	GAGCTGGACTAGAAT TGC ATGGCTACGGAAGTG
*At*PrR1_N98S	N98S F	CACT TCCGTAGCCA T AGC ATTCTAGTCCAGCTC
	N98S R	GAGCTGGACTAGAATGCTATGGCTACGGAAGTG
*At*PrR1_L46A	L46A F	AGGCCAGAGA TTGGT GCA GA AATCGAGAAAGTC
	L46A R	GACTTTCTCGATTTC TGC ACCAATCTCTGGCCT
*At*PrR1_L46V	L46V F	AGGCCAGAGA TTGGT GTT GA AATCGAGAAAGTC
	L46V R	GACTTTCTCGATTTC AAC ACCAATCTCTGGCCT
*At*PrR1_ L181F	L181F F	CAGAT GGTAACTTTA TTT CCTCCGAAAGAAAAA
	L181F R	TTTTTCTTTCGGAGGAAATAAAGTTACCATCTG
*At*PrR1_ L181A	L181A F	CAGAT GGTAACTTTA GCA CCTCCGAAAGAAAAA
	L181A R	TTTTTCTTTCGGAGG TGC TAAAGTTACCATCTG
*At*PrR2_N98S	N98S F	CACT TCCGCACCCA C AGC ATCCCCGTTCAGCTC
	N98S R	GAGCTGAACGGGGATGCTGTGGGTGCGGAAGTG
*At*PrR2_N98A	N98A F	CACT TCCGCACCCA C GCA ATCCCCGTTCAGCTC
	N98A R	GAGCTGAACGGGGATTGCGTGGGTGCGGAAGTG
*At*PrR2_V46L	V46L F	CGGCCGGAGA TTGGA CTG GA CATCGAGAAG GTG

	V46L R	CACCTTCTCGATGTC CAG TCCAATCTCCGGCCG
*At*PrR2_V46A	V46A F	CGGCCGGAGA TTGGA GCA GA CATCGAGAAG GTG
	V46A R	CACCTTCTCGATGTC TGC TCCAATCTCCGGCCG
*At*PrR2_F181I	F181I F	CAAAT GGGAACTTTG ATT CCTCCGAAAAACAAA
	F181I R	TTTGTTTTTCGGAGG AAT CAAAGTTCCCATTTG
*At*PrR2_ F181A	F181A F	CAAAT GGGAACTTTG GCA CCTCCGAAAAACAAA
	F181A R	TTTGTTTTTCGGAGG TGC CAAAGTTCCCATTTG
*At*PrR2_ F181L	F181L F	CAAAT GGGAACTTTG CTG CCTCCGAAAAACAAA
	F181L R	TTTGTTTTTCGGAGG CAG CAAAGTTCCCATTTG

Supplementary Table 2. Data collection and refinement statistics (molecular replacement)

	*Ii*PLR1_apo	*Ii*PLR1_NAP	*Ii*PLR1_NAP_+PIN	*Ii*PLR1_NAP_-PIN
**Data collection**				
Space group	C2	$P6_1$	$P6_1$	$P6_1$
Cell dimensions				
a, *b*, *c* (Å)	148.8, 242.5, 77.6	243.9, 243.9, 76.3	244.9, 244.9, 75.8	244.9, 244.9, 75.7
α, β, γ (°)	90, 111, 90	90, 90, 120	90, 90, 120	90, 90, 120
Resolution (Å)	31.19-2.69 (2.78-2.69) *	35.20-2.40 (2.49-2.40)	40.09-2.30 (2.39-2.30)	37.28-2.19 (2.27-2.19)
R_{sym} or R_{merge}	0.093 (0.764)	0.118 (0.755)	0.161 (1.516)	0.104 (0.694)
$I / \sigma I$	19.8 (2.3)	16.9 (2.7)	20.1 (2.1)	26.0 (4.6)
Completeness (%)	99.5 (99.5)	99.7 (98.9)	97.8 (96.7)	100.0 (100.0)
Redundancy	6.7 (6.6)	9.0 (8.2)	8.7 (8.7)	9.9 (9.5)
**Refinement**				
Resolution (Å)	31.19-2.69 (2.78-2.69)	35.20-2.40 (2.49-2.40)	40.09-2.30 (2.39-2.30)	37.28-2.19 (2.27-2.19)
No. reflections	70440 (6742)	98861 (8674)	111977 (10853)	133266 (13177)
R_{work} / R_{free}	0.1889 / 0.2303	0.2252 / 0.2603	0.2150 / 0.2367	0.1864 / 0.2150
No. atoms	13870	14395	14325	15035
Protein	1744	1746	1726	1726
Ligand/ion		288	444	444
Water	91	686	855	1549
B-factors	66.09	36.00	48.40	32.82
Protein	66.13	35.85	48.43	32.12
Ligand/ion		37.42	49.67	34.65
Water	60.34	39.97	45.87	40.59
R.m.s. deviations				
Bond lengths (Å)	0.011	0.011	0.011	0.010
Bond angles (°)	1.41	1.36	1.10	1.07

*Values in parentheses are for highest-resolution shell.

Supplementary Table 3. Data collection and refinement statistics (molecular replacement)

	*Ii*PLR1_NAP_-LAR	*Ii*PLR1_NAP_+SEC	*Ii*PLR1_NAP_-SEC	*At*PrR1_apo
**Data collection**				
Space group	$P6_1$	$P6_1$	$P6_1$	$P2_12_12_1$
Cell dimensions				
a, *b*, *c* (Å)	244.4, 244.4, 75.6	243.6, 243.6, 76.0	244.4, 244.4, 76.0	81.7, 134.9, 142.0
α, β, γ (°)	90, 90, 120	90, 90, 120	90, 90, 120	90, 90, 90
Resolution (Å)	40.87-2.20 (2.28-2.20)	32.17-2.30 (2.38-2.30)	39.99-2.60 (2.69-2.60)	39.27-2.80 (2.90-2.80)
R_{sym} or R_{merge}	0.137 (0.858)	0.182 (1.408)	0.191 (1.368)	0.152 (1.283)
$I / \sigma I$	24.1 (2.3)	18.1 (2.2)	16.3 (2.1)	19.3 (2.0)
Completeness (%)	100.0 (100.0)	99.8 (99.2)	100.0 (100.0)	99.9 (100.0)
Redundancy	7.4 (7.2)	9.9 (9.1)	10.4 (9.8)	12.1 (12.0)
**Refinement**				
Resolution (Å)	40.87-2.20 (2.28-2.20)	32.17-2.30 (2.38-2.30)	39.99-2.60 (2.69-2.60)	39.27-2.80 (2.90-2.80)
No. reflections	130415 (12796)	114525 (11189)	80035 (7931)	39296 (3860)
R_{work} / R_{free}	0.2187 / 0.2476	0.2142 / 0.2555	0.2079 / 0.2388	0.2160 / 0.2750
No. atoms	14826	14708	14214	9564
Protein	1751	1730	1748	1215
Ligand/ion	444	444	444	
Water	1131	1185	542	
B-factors	36.80	36.75	43.16	67.30
Protein	36.50	36.26	43.10	67.30
Ligand/ion	35.44	44.13	44.79	
Water	43.77	41.17	44.36	
R.m.s. deviations				
Bond lengths (Å)	0.010	0.011	0.012	0.012
Bond angles (°)	1.18	1.19	1.42	1.77

*Values in parentheses are for highest-resolution shell.

Supplementary Table 4. Data collection and refinement statistics (molecular replacement)

	*At*PrR1_NAP	*At*PrR1_NAP_+PIN	*At*PrR1_NAP_-PIN	*At*PrR1_NAP_+LAR
**Data collection**				
Space group	$P2_1$	$P2_1$	C2	$P2_1$
Cell dimensions				
a, *b*, *c* (Å)	74.3, 144.3, 77.3	74.2, 144.7, 77.0	211.0, 128.7, 105.3	73.9, 143.7, 76.1
α, β, γ (°)	90, 116, 90	90, 116, 90	90, 92, 90	90, 116, 90
Resolution (Å)	32.44-1.96 (2.03-1.96)	33.90-2.00 (2.07-2.00)	10.09-2.52 (2.60-2.52)	33.18-1.80 (1.86-1.80)
R_{sym} or R_{merge}	0.099 (0.977)	0.092 (0.719)	0.170 (0.939)	0.085 (0.857)
$I / \sigma I$	17.4 (2.0)	24.2 (2.2)	11.7 (1.6)	27.9 (2.0)
Completeness (%)	99.5 (99.9)	89.2 (100.0)	99.7 (99.5)	99.6 (99.9)
Redundancy	6.1 (5.7)	3.7 (3.8)	4.0 (4.1)	3.6 (3.7)
**Refinement**				
Resolution (Å)	32.44-1.96 (2.03-1.96)	33.90-2.00 (2.07-2.00)	10.09-2.52 (2.60-2.52)	33.18-1.80 (1.86-1.80)
No. reflections	100650 (8293)	86455 (9547)	92981 (8665)	129807 (11685)
R_{work} / R_{free}	0.1816 / 0.2225	0.1958 / 0.2326	0.2215 / 0.2519	0.1839 / 0.2184
No. atoms	11257	10609	15236	11025
Protein	1244	1243	1865	1243
Ligand/ion	192	296	444	296
Water	1469	866	637	1286
B-factors	30.25	41.17	42.21	35.57
Protein	29.35	41.11	42.22	34.74
Ligand/ion	23.99	36.53	42.51	31.79
Water	38.09	44.61	41.25	44.88
R.m.s. deviations				
Bond lengths (Å)	0.008	0.009	0.011	0.007
Bond angles (°)	1.04	1.18	1.09	1.02

*Values in parentheses are for highest-resolution shell.

Supplementary Table 5. Data collection and refinement statistics (molecular replacement)

	*At*PrR1_NAP_-LAR	*At*PrR1_NAP_-SEC	*At*PrR2_apo	*At*PrR2_NAP_+PIN
**Data collection**				
Space group	C2	$P2_1$	P1	$C222_1$
Cell dimensions				
a, *b*, *c* (Å)	211.6, 129.5, 106.3	74.1, 143.2, 77.1	44.3, 57.9, 71.6	78.0, 90.5, 117.6
α, β, γ (°)	90, 91, 90	90, 116, 90	107, 96, 113	90, 90, 90
Resolution (Å)	47.11-2.44 (2.53-2.44)	38.40-1.98 (2.05-1.98)	34.05-2.00 (2.07-2.00)	39.00-1.59 (1.65-1.59)
R_{sym} or R_{merge}	0.124 (1.082)	0.121 (1.060)	0.115 (0.422)	0.102 (0.475)
$I / \sigma I$	22.6 (1.9)	25.5 (2.1)	24.3 (6.0)	20.7 (3.5)
Completeness (%)	100.0 (100.0)	100.0 (100.0)	97.6 (97.6)	99.7 (99.5)
Redundancy	7.5 (7.6)	7.5 (7.6)	3.6 (3.9)	12.9 (11.4)
**Refinement**				
Resolution (Å)	47.11-2.44 (2.53-2.44)	38.40-1.98 (2.05-1.98)	34.05-2.00 (2.07-2.00)	39.00-1.59 (1.65-1.59)
No. reflections	106056 (10355)	99395 (9769)	39960 (3909)	55656 (5475)
R_{work} / R_{free}	0.2058 / 0.2326	0.1739 / 0.2097	0.1851 / 0.2325	0.1536 / 0.1756
No. atoms	15502	10893	4831	3035
Protein	1865	1244	568	312
Ligand/ion	444	296		74
Water	903	1141	378	586
B-factors	40.09	38.04	25.73	17.27
Protein	40.01	37.32	25.07	14.51
Ligand/ion	38.47	44.90		10.26
Water	44.16	43.99	33.50	31.48
R.m.s. deviations				
Bond lengths (Å)	0.010	0.009	0.008	0.011
Bond angles (°)	1.19	1.19	0.92	1.28

*Values in parentheses are for highest-resolution shell.
