# Supplementary material for: Structure-based engineering of substrate specificity for pinoresinol-lariciresinol reductases
Source: Nat Commun. 2021 May 14;12:2828. doi: 10.1038/s41467-021-23095-y (PMC8121951; doi:10.1038/s41467-021-23095-y)
Supplement: Supplementary file 4 — Description of additional supplementary files [file 41467_2021_23095_MOESM4_ESM.docx]

Description of additional supplementary information

Title: Supplementary Movie 1.

Description: A movie showing how PLR and PrR change conformation throughout the catalytic process.
