# Supplementary material for: Structure-based engineering of substrate specificity for pinoresinol-lariciresinol reductases
Source: Nat Commun. 2021 May 14;12:2828. doi: 10.1038/s41467-021-23095-y (PMC8121951; doi:10.1038/s41467-021-23095-y)
Supplement: Supplementary file 2 — Peer Review File [file 41467_2021_23095_MOESM2_ESM.pdf]

REVIEWER COMMENTS

Reviewer #1 (Remarks to the Author):

To understand the substrate selectivities of pinoresinol-lariciresinol reductases, the authors solved 16 crystal structures and performed detailed site-mutagenesis experiments. The results revealed that the entrance and orientation of the substrate in AtPrR1/2 is more tightly controlled than LiPLR2. The data also showed that substrate specificity of PLR/PrR could be switched by structure-based engineering. The structure information from this work also provide insights how PLRs have strict enantiospecificities. The manuscript is very well organized and written in succint English.I think it should be accepted after minor revision.
All statistics analysis including the data collection and structure refinement are valid to me.

Minor suggestion:
The final working model in Figure 5 proposed by the authors provide insights of how PLR and PrR function in catalysis. It will be nice to use several structures solved in this work to generate one or two movie to demonstrate the detailed process how the enzyme undergoes conformational change to make the substrate binding and catalysis happen.

Reviewer #2 (Remarks to the Author):

This is a timely and truly informative manuscript that advances the field. The structural findings and conclusions are certainly supported by the the data supportive. Of concern, is the hypothesis centered about structural dynamics such as "We further deduced that any residue in PLR/PrRs around the hydrophobic groove or affecting homodimerization may impact the conformation of the active site, thereby dictating substrate selectivity" and "Taking structure and enzymology data together, we proposed a three-step catalytic
193 mechanism for PLR based on its homodimerization. First, the monomeric apo PLR recruits free NADPHs through the very flexible β4 loop. Second, upon homodimerization, pinoresinol binds to one protomer via the substrate-binding groove, and the other protomer helps stabilize the substrate. Subsequently, pinoresinol receives H: from NADPH and be reduced to lariciresinol released later. Third, free lariciresinol is bound by another reductive PLR molecule and fixed by another198 homodimer, and then the lariciresinol is reduced to secoisolariciresinol ..."

Given the experimental data provided, these speculative claims need to be supported with molecular modeling

Reviewer #3 (Remarks to the Author):

This manuscript describes the structural elucidation of several forms of pinoresinol-lariciresinol reductases and the examination of their ability to reduce each of their substrates through site directed mutagenesis. Although I should state up front that I am not a structural biologist, in general the experiments look well done for the most part.
My major critiques have to do with 1) the framing of the engineering aspect of the paper, 2) whether the results obtained actually amount to "switching substrate specificity" and 3) whether the claim of switching substrate specificity can even be made in the absence of a full kinetic characterization of the enzyme (see points below on the length of the assays used and a potential lack of linearity).
With regard to point 1 above, the authors don't introduce the concept anywhere in the paper that their actual objective is to eliminate the second reaction that PLR is capable of performing. Instead, they include statements like "In our present work, structure-guided mutagenesis successfully switched the substrate specificity of IiPLR1, leading to overproduction of lariciresinol by E. coli." It would be much more straightforward to say that they reduced secoisolariciresinol production. These issues may be

obvious to experts in the field, but it was not obvious to me until quite late in the paper. This concept could easily be addressed in the Introduction.

With regard to points 2 and 3, I must first say that I don't think that the authors provide enough data (full kinetic analysis) to support their claims, and also that I'm not sure that this really amounts to switching the substrate specificity of the enzyme in a substantial way. Has the enzyme's activity towards lariciresinol even been altered? I don't think we can tell with the data provided.

For the model described at the bottom of page 7 the authors state "Second, upon homodimerization, pinoresinol binds to one protomer via the substrate-binding groove, and the other protomer helps stabilize the substrate. Subsequently, pinoresinol receives H: from NADPH and be reduced to lariciresinol released later. Third, free lariciresinol is bound by another reductive PLR molecule and fixed by another homodimer, and then the lariciresinol is reduced to secoisolariciresinol and finally released (Fig. 5)." What is the evidence that lariciresinol has to be released before a subsequent reductive step?

The phylogenetic tree in Fig. 1B has poorly supported bootstraps and is thus unreliable. Furthermore, the authors do not state what program was used to generate it. This tree should be regenerated with a reliable program and the method for doing so should be clearly described. In addition, there is no explanation of what the protein names mean in the figure legend.

In the Methods section on line 323, the authors state that assays were run for 30 minutes, and yet for the determination of kinetic constants, assays were run for only 5 minutes, with the authors stating that this was in the linear kinetic range. Does this mean that 30 minutes was not? Further to this point, the extent of substrate conversion shown in Figure 4 is very high and almost certainly does not represent first order kinetics. As a result, I think that these results may be misleading.

Figure 5B is not very informative and could easily be deleted. Again the figure is not fully explained by its legend. What are the blue dumbbell-shaped objects meant to represent?

What kind of replication was performed for Figure 6 and what is represented by the error bars (standard deviation, standard error)? The error bars are so small that it is tempting to speculate that the only replicates were technical replicates rather than biological replicates, which would be unacceptable.

The authors state "Consistent with the data for IiPLR1, AtPrR1 178 mutants L46A and L46V could enhance the conversion rate of lariciresinol and partially reduce that of secoisolariciresinol, which confirmed the importance of these two sites in catalysis." Does it really? Depends on your definition of catalysis I guess, but the mutation of these residues could, for example, facilitate product release which happens after catalysis.

Why doesn't Figure 6 show secoisolariciresinol levels. Isn't the elimination of this product one of the points of this paper?

Minor points

The writing of the manuscript is quite good for the most part, although there are some errors that could be corrected by the journal staff prior to publication. On the other hand, most figure legends are not nearly detailed enough.

Among those that should be corrected prior to resubmission include:

Line 47 "specifically catalyze lariciresinol" and Line 190-191 "gained the activity to catalyze lariciresinol (Fig. 4c)." An enzyme cannot catalyze a molecule. It must catalyze its conversion to something else.

The sentence that reads "To determine the catalytic mechanism underlying this difference in substrate specificity, we confirmed the relative lack of activity for AtPrR1/2 (Fig. 1) and then carried out a structure analysis of IiPLR1, AtPrR1, and AtPrR2." is odd in the way that it uses catalytic mechanism and substrate specificity. In my opinion, catalytic mechanism is something that is often quite separate from substrate specificity. The sentence would be improved simply by deleting the word "catalytic".

With regard to the sentence "In contrast to IiPLR1_NAP_+PIN, the β4 loops of AtPrR1_NAP_+PIN and AtPrR2_NAP_+PIN can be clearly identified (Fig. 3 and Supplementary Fig. 4).", the beta4 loop is only shown in Figure 2 and at relatively low magnification. It shoud be highlighted in Figure 3 if it is important enough to mention in Results.

In the section entitled "Mutagenesis-based alteration of the substrate selectivity" a lot of data is

discussed before Figure 4 is finally mentioned on line 187. Mention of this figure should be moved to earlier in the paragraph.
The axis label for Figure 4B reads coversion instead of conversion.
The axis label for Figure 6 reads yeild instead of yield.

Our responses to each of the comments and suggestions are listed below.

Reviewer #1:

Minor suggestion:

The final working model in Figure 5 proposed by the authors provide insights of how PLR and PrR function in catalysis. It will be nice to use several structures solved in this work to generate one or two movie to demonstrate the detailed process how the enzyme undergoes conformational change to make the substrate binding and catalysis happen.

The authors' response: Thanks for the reviewer's suggestion. We have attached our movie (Supplementary Movie 1) showing how the enzymes change conformation throughout a single round of catalysis.

Reviewer #2:

This is a timely and truly informative manuscript that advances the field. The structural findings and conclusions are certainly supported by the the data supportive. Of concern, is the hypothesis centered about structural dynamics such as "We further deduced that any residue in PLR/PrRs around the hydrophobic groove or affecting homodimerization may impact the conformation of the active site, thereby dictating substrate selectivity" and "Taking structure and enzymology data together, we proposed a three-step catalytic mechanism for PLR based on its homodimerization. First, the monomeric apo PLR recruits free NADPHs through the very flexible β4 loop. Second, upon homodimerization, pinoresinol binds to one protomer via the substrate-binding groove, and the other protomer helps stabilize the substrate. Subsequently, pinoresinol receives H: from NADPH and be reduced to lariciresinol released later. Third, free lariciresinol is bound by another reductive PLR molecule and fixed by another198 homodimer, and then the lariciresinol is reduced to secoisolariciresinol ..."

Given the experimental data provided, these speculative claims need to be supported with molecular modeling

The authors' response: We understand the reviewer's concerns. The previously reported Lys138 in *Tp*PLR1, which corresponds to Lys144 in *Ii*PLR1, was identified as the general base for catalysis because K138A lacks the ability to convert pinoresinol (ref. 17). Here, we found that Lys144 formed direct hydrogen bonds with $NADP^+$ and was away from the furan ring of each substrate. Lys144 may not participate in catalysis directly but rather affects the stability of NADPH, thus affecting catalysis. A recent study reported that the L174I mutant of *Cs*PLR1 cannot reduce pinoresinol and specifically catalyze lariciresinol (ref. 16), and this residue is near the hydrophobic groove.

Concerning the description of dimerization, there was a mistake in our original manuscript. The original description "First, the monomeric apo PLR recruits free NADPHs through the very flexible β4 loop. Second, upon homodimerization, pinoresinol binds to one protomer via the substrate-binding groove, and the other protomer helps stabilize the substrate" has been revised to "First, the protomers of dimeric PLRs recruit free NADPH through the very flexible β4 loop. Second, pinoresinol binds into one protomer via the substrate-binding groove, and the other protomer of the homodimer helps stabilize the substrate." Refer to this revised text in lines 197–199 of the revised manuscript. In addition, we created a movie (molecular modeling, Supplementary Movie 1) showing how the enzymes change conformation throughout the catalytic process.

Reviewer #3:

Comment 1: My major critiques have to do with 1) the framing of the engineering aspect of the paper, 2) whether the results obtained actually amount to "switching substrate specificity" and 3) whether the claim of switching substrate specificity can even be made in the absence of a full kinetic characterization of the enzyme (see points below on the length of the assays used and a potential lack of linearity).

With regard to point 1 above, the authors don't introduce the concept anywhere in the paper that their actual objective is to eliminate the second reaction that PLR is capable of performing. Instead, they include statements like "In our present work, structure-guided mutagenesis successfully switched the substrate specificity of

IiPLR1, leading to overproduction of lariciresinol by E. coli." It would be much more straightforward to say that they reduced secoisolariciresinol production. These issues may be obvious to experts in the field, but it was not obvious to me until quite late in the paper. This concept could easily be addressed in the Introduction.

With regard to points 2 and 3, I must first say that I don't think that the authors provide enough data (full kinetic analysis) to support their claims, and also that I'm not sure that this really amounts to switching the substrate specificity of the enzyme in a substantial way. Has the enzyme's activity towards lariciresinol even been altered? I don't think we can tell with the data provided.

The authors' response: We thank the reviewer for these important comments. With regard to point 1, we have revised the original sentence "structure-guided mutagenesis successfully switched the substrate specificity of *Ii*PLR1, leading to overproduction of lariciresinol by *E. coli*" to "structure-guided mutagenesis successfully switched the substrate specificity of *Ii*PLR1, leading to overproduction of lariciresinol and reduced production of secoisolariciresinol by *E. coli*" (lines 276 and 277). Meanwhile, we have emphasized the concept that one of our objectives for the mutagenesis study was to eliminate the second reaction of PLR. See lines 63–66 in the revised Introduction, and lines 181, 209, and 223–227 in the Results.

With regard to points 2 and 3, we understand the reviewer's concerns and have added a full kinetic characterization for the substrates pinoresinol and lariciresinol (Table 2), and the results demonstrate that our mutagenesis approach caused a switch in the substrate specificity of *Ii*PLR1 and altered its catalytic efficiency for both pinoresinol and lariciresinol, and especially the second reaction was eliminated (lines 174–181). With regard to the length of the assays and linearity, refer to our detailed response to **Comment 4**.

Comment 2: For the model described at the bottom of page 7 the authors state "Second, upon homodimerization, pinoresinol binds to one protomer via the substrate-binding groove, and the other protomer helps stabilize the substrate. Subsequently, pinoresinol receives H: from NADPH and be reduced to lariciresinol

released later. Third, free lariciresinol is bound by another reductive PLR molecule and fixed by another homodimer, and then the lariciresinol is reduced to secoisolariciresinol and finally released (Fig. 5)." What is the evidence that lariciresinol has to be released before a subsequent reductive step?

The authors' response: Our inference is that lariciresinol has to be released before the subsequent reductive step, based on analysis of the crystal structures. In our $NADP^+$-bound structures, Phe166 forms a π-π stack with the catalytically active nicotinamide group of $NADP^+$, and this interaction is important for stabilization of $NADP^+$ (mainly the active nicotinamide group). After catalyzing the conversion of pinoresinol to lariciresinol, both lariciresinol and oxidized $NADP^+$ should be released. Of course, the enzyme must recruit a reductive NADPH to carry out another reaction; if lariciresinol cannot be released, however, then it would be very difficult to release $NADP^+$ and bind a new NADPH. Therefore, we concluded that the two reactions carried out by PLR were not continuous but rather divided into two steps, and thus we propose that lariciresinol must be released before the subsequent reductive step.

We have revised the manuscript to clarify the description of the catalytic process (lines 196–203), and we added a movie (Supplementary Movie 1) to show how the enzymes undergo conformational changes during catalysis.

Comment 3: The phylogenetic tree in Fig. 1B has poorly supported bootstraps and is thus unreliable. Furthermore, the authors do not state what program was used to generate it. This tree should be regenerated with a reliable program and the method for doing so should be clearly described. In addition, there is no explanation of what the protein names mean in the figure legend.

The authors' response: We have reconstructed the phylogenetic tree (Fig. 1b) and added both the method and gene information (lines 284–292) in the revised manuscript.

Comment 4: In the Methods section on line 323, the authors state that assays were

run for 30 minutes, and yet for the determination of kinetic constants, assays were run for only 5 minutes, with the authors stating that this was in the linear kinetic range. Does this mean that 30 minutes was not? Further to this point, the extent of substrate conversion shown in Figure 4 is very high and almost certainly does not represent first order kinetics. As a result, I think that these results may be misleading.

The authors' response: We understand the reviewer's concerns and have revised the manuscript accordingly (see line 349). Indeed, the extent of substrate conversion shown in Figure 4 (assays were run for 30 minutes) is very high, implying that at this point we could no longer measure Vo for the determination of kinetic constants. To obtain reliable data for kinetics, the substrate should not be consumed by more than 10%. Therefore, to obtain accurate kinetics data, the assays were performed again, but this time for only 5 minutes, thereby ensuring that substrate consumption was $\leqslant$10%. In addition, considering the further conversion of the intermediate product lariciresinol into secoisolariciresinol, we used the consumption of substrate to determine kinetics. A full kinetic characterization of the enzyme was also added (see Table 2 and lines 174–181).

Comment 5: Figure 5B is not very informative and could easily be deleted. Again the figure is not fully explained by its legend. What are the blue dumbbell-shaped objects meant to represent?

The authors' response: The purpose of Figure 5B was to give readers an intuitive understanding of the work. We thank the reviewer for the suggestion and have revised the manuscript to clarify the catalytic processes (lines 196–203). Also, as noted in our response to 'Comment 2', we added a movie that clarifies the overall catalytic process (Supplementary Movie 1). The blue dumbbell-shaped objects represent switches composed of β4 loops, and we have updated the figure legend (lines 504–506) accordingly.

Comment 6: What kind of replication was performed for Figure 6 and what is represented by the error bars (standard deviation, standard error)? The error bars are

so small that it is tempting to speculate that the only replicates were technical replicates rather than biological replicates, which would be unacceptable.

The authors' response: Thanks to the reviewer. We have repeated the experiment and revised the methods (Line 353–369) and results (Line 209-230) accordingly. For Figure 6, bioconversion was carried out independently with three biological replicates, and error bars represent standard deviation. To enable accurate assessment of *Ii*PLR and its mutants, the OD600 of *E. coli* cells in culture was normalized to the same level to avoid the effect of cell concentration on enzyme activity (lines 364 and 366). This normalization may have reduced the size of the error bars but did not affect the comparison of enzymatic activity.

Comment 7: The authors state "Consistent with the data for IiPLR1, AtPrR1 178 mutants L46A and L46V could enhance the conversion rate of lariciresinol and partially reduce that of secoisolariciresinol, which confirmed the importance of these two sites in catalysis." Does it really? Depends on your definition of catalysis I guess, but the mutation of these residues could, for example, facilitate product release which happens after catalysis.

The authors' response: Residue L46 of each of *Ii*PLR1 and *At*PrR1/2 not only helps stabilize the substrate during catalysis but also affects product release. We have revised the original text "which confirmed the importance of these two sites in catalysis" as follows: "which confirmed the importance of these two sites in substrate binding and product release thus in catalysis". See lines 183‒184 in the revised manuscript.

Comment 8: Why doesn't Figure 6 show secoisolariciresinol levels. Isn't the elimination of this product one of the points of this paper?

The authors' response: We appreciate this insightful comment. The secoisolariciresinol levels have been added in the revised Figure 6.

Comment 9: The writing of the manuscript is quite good for the most part, although there are some errors that could be corrected by the journal staff prior to publication. On the other hand, most figure legends are not nearly detailed enough.

The authors' response: We have updated the figure legends (lines 462-509) and invited a native English speaker to further polish the language. The revision was highlighted in red color in the revised manuscript.

Comment 10: Line 47 "specifically catalyze lariciresinol" and Line 190-191 "gained the activity to catalyze lariciresinol (Fig. 4c)." An enzyme cannot catalyze a molecule. It must catalyze its conversion to something else.

The authors' response: Thanks to the reviewer. We have revised these sentences accordingly. See lines 45, 127-129, 194–195, 226-227 and 247 in the revised manuscript.

Comment 11: The sentence that reads "To determine the catalytic mechanism underlying this difference in substrate specificity, we confirmed the relative lack of activity for AtPrR1/2 (Fig. 1) and then carried out a structure analysis of IiPLR1, AtPrR1, and AtPrR2." is odd in the way that it uses catalytic mechanism and substrate specificity. In my opinion, catalytic mechanism is something that is often quite separate from substrate specificity. The sentence would be improved simply by deleting the word "catalytic".

The authors' response: Thanks to the reviewer. We have revised this sentence following the reviewer's suggestion. See lines 135-136 in the revised manuscript.

Comment 12: With regard to the sentence "In contrast to IiPLR1_NAP_+PIN, the β4 loops of AtPrR1_NAP_+PIN and AtPrR2_NAP_+PIN can be clearly identified (Fig. 3 and Supplementary Fig. 4).", the beta4 loop is only shown in Figure 2 and at relatively low magnification. It shoud be highlighted in Figure 3 if it is important enough to mention in Results.

The authors' response: We have updated Figure 3 and its figure legend (lines

491-497) following the reviewer’s suggestion.

Comment 13: In the section entitled “Mutagenesis-based alteration of the substrate selectivity” a lot of data is discussed before Figure 4 is finally mentioned on line 187. Mention of this figure should be moved to earlier in the paragraph.

The authors’ response: Thanks to the reviewer, and we have revised it following the reviewer’s suggestion. See lines 173, 183, 186, and 189 in the revised manuscript.

Comment 14: The axis label for Figure 4B reads coversion instead of conversion. The axis label for Figure 6 reads yeild instead of yield.

The authors’ response: We have corrected these mistakes in the revised manuscript (Figures 4b and 6).

REVIEWERS' COMMENTS

Reviewer #3 (Remarks to the Author):

The authors have done a very thorough job in responding to all of my comments.
